# Decomposition mechanism of α-alkoxyalkyl-hydroperoxides in the liquid phase: temperature dependent kinetics and theoretical calculations†

**DOI:** 10.1039/d1ea00076d

**Published:** 2022-01-17

**Authors:** Mingxi Hu, Kunpeng Chen, Junting Qiu, Ying-Hsuan Lin, Kenichi Tonokura, Shinichi Enami

**Affiliations:** a Graduate School of Frontier Sciences, The University of Tokyo 5-1-5 Kashiwanoha Kashiwa 277-8563 Japan; b Department of Environmental Sciences, University of California Riverside California 92521 USA; c National Institute for Environmental Studies 16-2 Onogawa Tsukuba 305-8506 Japan enami.shinichi@nies.go.jp +81-29-850-2770

## Abstract

Organic hydroperoxides (ROOHs) play key roles in the atmosphere as a reactive intermediate species. Due to the low volatility and high hydrophilicity, ROOHs are expected to reside in atmospheric condensed phases such as aerosols, fogs, and cloud droplets. The decomposition mechanisms of ROOHs in the liquid phase are, however, still poorly understood. Here we report a temperature-dependent kinetics and theoretical calculation study of the aqueous-phase decompositions of C_12_ or C_13_ α-alkoxyalkyl-hydroperoxides (α-AHs) derived from ozonolysis of α-terpineol in the presence of 1-propanol, 2-propanol, and ethanol. We found that the temporal profiles of α-AH signals, detected as chloride-adducts by negative ion electrospray mass spectrometry, showed single-exponential decay, and the derived first-order rate coefficient *k* for α-AH decomposition increased as temperature increased, *e.g.*, *k*(288 K) = (5.3 ± 0.2) × 10^−4^ s^−1^, *k*(298 K) = (1.2 ± 0.3) × 10^−3^ s^−1^, *k*(308 K) = (2.1 ± 1.4) × 10^−3^ s^−1^ for C_13_ α-AHs derived from the reaction of α-terpineol Criegee intermediates with 1-propanol in the solution at pH 4.5. Arrhenius plot analysis yielded an activation energy (*E*_a_) of 12.3 ± 0.6 kcal mol^−1^. *E*_a_ of 18.7 ± 0.3 and 13.8 ± 0.9 kcal mol^−1^ were also obtained for the decomposition of α-AHs (at pH 4.5) derived from the reaction of α-terpineol Criegee intermediates with 2-propanol and with ethanol, respectively. Based on the theoretical kinetic and thermodynamic calculations, we propose that a proton-catalyzed mechanism plays a central role in the decomposition of these α-AHs in acidic aqueous organic media, while water molecules may also participate in the decomposition pathways and affect the kinetics. The decomposition of α-AHs could act as a source of H_2_O_2_ and multifunctionalized species in atmospheric condensed phases.

## Abstract 2

The decomposition rates of organic hydroperoxides derived from ozonolysis of terpene-alcohol in the presence of short-chain alcohols in aqueous organic media is markedly increased as the temperature is increased.

## Graphical abstract

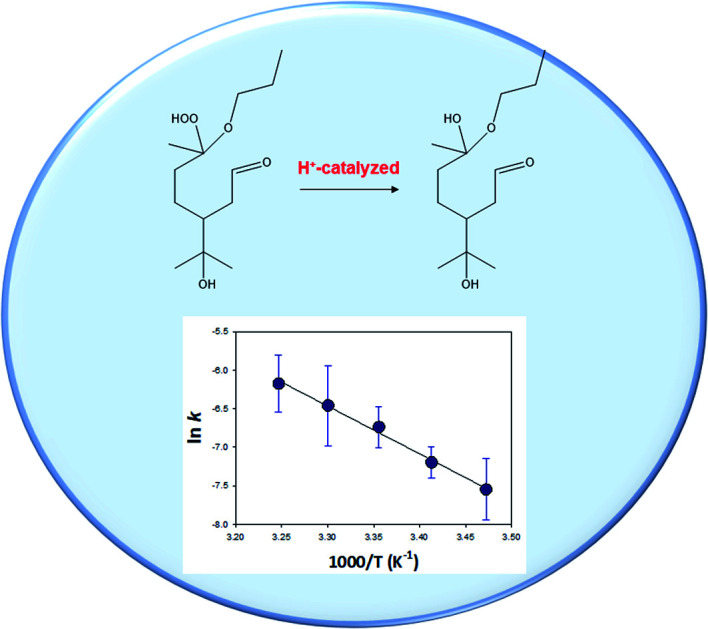

**Environmental significance**

The decomposition of organic hydroperoxides (ROOHs) contributes to the formation of H_2_O_2_ and multifunctionalized species in atmospheric condensed phases, which play central roles in modulating many atmospheric processes. Our results show that the stability of α-alkoxyalkyl-hydroperoxides (α-AHs) derived from ozonolysis of α-terpineol in the presence of short-chain alcohols in aqueous organic media is markedly increased as the temperature is decreased, implying that the lifetimes of α-AHs in aerosols are affected by local temperatures under different environmental conditions such as altitude, day/night, season, and weather. The derived *E*_a_ values for the decomposition of the α-AHs could be incorporated in atmospheric modeling.

## Introduction

Among a variety of reactive oxygen species, organic hydroperoxides (ROOHs) possessing single or multiple –OOH moieties (*e.g.*, highly oxygenated organic molecules, HOM) are a ubiquitous intermediate species formed by the oxidation of volatile organic compounds (VOCs).^1–5^ ROOHs are largely formed *via* two major atmospheric processes, that is, oxidations of VOCs initiated by OH-radical (RO_2_ chemistry) and ozone (Criegee chemistry). The former process involves the reaction RO_2_ + HO_2_ → ROOH + O_2_ (ref. 6) and intramolecular autoxidation of RO_2_ leading to the formation of HOM,^7^ that contain multiple –OOH groups. The latter process involves reactive carbonyl oxide zwitterions/biradicals, known as Criegee intermediates (CIs),^8^ generated by ozonolysis of unsaturated VOCs possessing C=C bond(s) such as biogenic terpenes. CIs can rapidly react with OH-containing species such as water, alcohols and carboxylic acids to form ROOHs in the gas-phase or liquid-phase and at the air–liquid interface.^8–10^ The reactions of CIs with ammonia, amine, HCl, H_2_S also produce ROOHs.^8^ Among the reaction partners of CIs, water (H_2_O)_*n*≥1_ is expected to be the most important reactant because of its predominant abundance in both the gas-phase and the condensed phases. At relative humidity (RH) = 50%, for example, the concentration of gaseous water dimer (H_2_O)_2_ reaches 3.0 × 10^14^ molecule per cm^3^ and it could be a dominant reactant for CH_2_OO (the smallest CI), which converts to hydroxymethyl hydroperoxide within 0.5 ms.^11^ The hydration of CIs produces α-hydroxyalkyl-hydroperoxides (α-HHs) possessing –OOH and –OH moieties.

Because of the low volatilities and high hydrophilicities, these ROOHs are expected to be taken up into atmospheric condensed phases. We have found that the α-HHs in aqueous phase generated by ozonolysis of terpenes decompose into functionalized geminal diols and the decompositions occur with lifetimes (*τ*_1/e_) that depend on the water content, the pH, and the temperature of the medium as well as the chemical structure of the α-HH.^1,12–15^ Recently, we investigated the fates of α-alkoxyalkyl-hydroperoxides (α-AHs), an important ROOH derived from the reaction of CIs with alcohols, in the liquid phase.^16^ α-AHs possess –OOH and –OR′ groups attached to the same carbon-atom. Given that atmospheric mixing ratios of short-chain alcohols exceed a few parts per billion by volume (ppbv) at forest sites,^12,15,16^ CIs could be partly converted by alcohols into α-AHs under specific conditions (*e.g.*, at low relative humidity). In addition to the direct source, alcohols are formed during the oxidation of VOCs, such as incorporation of OH into C=C double bonds.^17,18^ α-AHs can be formed by ozonolysis of VOCs with alcohols not only in the gas-phase but also at the air–water interface.^19,20^ Interestingly, β-caryophyllene/α-humulene CIs can react with saccharides, a major component of biomass burning particles, to form α-AHs at the air–water interface.^19^ Previously, we successfully detected C_11_–C_13_ α-AHs, as their chloride-adducts by on-line electrospray mass spectrometry, generated from ozonolysis of C_10_ monoterpene-alcohol α-terpineol (α-Tp) in solutions with the presence of C_1_–C_3_ alcohols. α-Tp is a representative monoterpene alcohol that is emitted from plants and household products.^21,22^ Among terpenes, we selected α-Tp because of its high solubility in neat water (∼10^3^ times more soluble than α-pinene) and its structural similarity to other monoterpenes such as α-pinene and d-limonene.^23^ We found the decay rate of C_13_ α-AHs derived from the α-Tp CIs + 1-propanol reaction increased as pH decreased from pH 5.9 to 3.8, implying an acid-catalyzed decomposition mechanism.^16^ A primary decomposition product of α-AHs in an acidic aqueous solution was found to be a hemiacetal, that possesses an –OH and an –OR′ attached to the same carbon-atom. However, the decomposition mechanisms of α-AHs in the liquid phase are not fully understood yet.

**Fig. 1:**
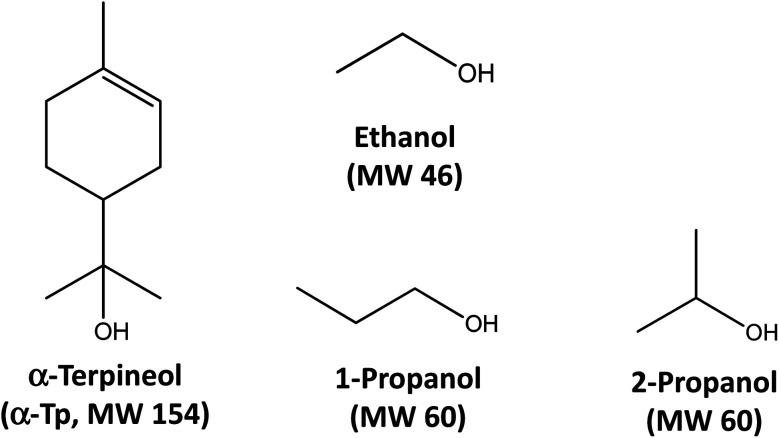
Chemical structures and molecular weights (MWs) of α-terpineol, ethanol, 1-propanol, and 2-propanol.

Here, we extend our research by performing new experiments of α-AH decomposition in aqueous organic solutions at different temperatures, in addition to theoretical calculations for elucidation of the decomposition mechanism for details. We measured temporal profiles of α-AHs in solutions at *T* = 288–308 K derived from ozonolysis of α-Tp in the presence of C_3_ 1-propanol, 2-propanol or C_2_ ethanol (Fig. 1). Direct monitoring of the temporal profiles of α-AHs enabled us to derive rate coefficients (*k*) at *T* = 288–308 K and activation energies (*E*_a_) of α-AHs decomposition in the liquid phase for the first time, that will be useful for atmospheric modeling and interpretation of results obtained from field measurements.

Theoretical calculations of Gibbs free energy profiles and reaction rate coefficients were also performed to evaluate the role of proton (H^+^)-catalyzed and water-catalyzed decomposition mechanisms by taking α-AHs in 1-propanol:water and 2-propanol:water solutions as the model compounds. Results of the theoretical calculations may further shed light on the general decomposition mechanism for ROOHs in atmospheric condensed phases.

## Experimental section

Fig. S1† shows the schematic procedure for preparing α-AHs in alcohol : water solutions. 2 mM α-Tp and 0.4 mM NaCl were dissolved in 10 mL of neat alcohol in a glass vial (25 mL) in a Peltier-type circulating water bath (AS ONE corporation, CTB-1). Aqueous O_3_ solutions were prepared by sparging 10 mL of water in a 25 mL vial (kept in the water bath in advance) for 7–30 s with O_3_(g) generated by means of a commercial ozonizer (KSQ-050, Kotohira, Japan) fed with ultra-high-purity O_2_(g) (>99.999%). The output gases from the ozonizer were carried to the vial by means of Teflon tubing (3 mm i.d.) at a flow rate of 1 L min^−1^ (regulated by a digital mass-flow controller, Horiba STEC). The initial O_3_ concentrations in the solutions, [O_3_(aq)]_0_, were 0.06 ± 0.01 mM, determined with a UV–vis spectrometer (Agilent 8453) using the reported O_3_ molar extinction coefficient at 258 nm (*ε*_258 nm_ = 3840 M^−1^ cm^−1^ in water^24^).

Ozonolysis reactions were initiated by mixing the α-Tp + NaCl in an alcohol solution and the O_3_ in an aqueous solution (10 mL each) in a 25 mL glass vial in the water bath. The temperature of the reactants and reaction mixtures were maintained within ±1.0 K. Then, hydrochloric acid (HCl) was added to the mixtures for experiments at pH of 4.0 or 4.5. The pH values of the α-Tp + NaCl + HCl solutions were measured with a calibrated pH meter (LAQUA F-74, Horiba) in separate experiments. The [α-Tp]_0_/[O_3_(aq)]_0_ ratio was kept constant at ≈17, ensuring that O_3_ is consumed exclusively by α-Tp ([α-Tp]_0_ = 1 mM, *k* = 9.9 × 10^6^ M^−1^ s^−1^) within a lifetime *τ*_1/e_ of ∼0.1 ms.^25^ The reaction mixtures were immediately injected at 100 μL min^−1^*via* a glass syringe (5 mL, covered with aluminum foil) and a syringe pump (Pump 11 Elite, Harvard apparatus) into an electrospray mass spectrometer (ESMS, Agilent 6130 Quadrupole LC/MS Electrospray System housed at the National Institute for Environmental Studies, Tsukuba, Japan).

Adding sub-millimolar amounts of NaCl to the sample solutions enables us to detect Cl^−^ adducts of specific ROOHs, including α-HHs and α-AHs, and other chemical species by ESMS. We demonstrated that species possessing at least three functional groups, including a peroxide (–OOH/–OOR), an alcohol (–OH), or a ketone (–RC=O) are MS-detectable as the Cl^−^ adducts.^1,12,13,26^ The temporal profiles of the ion signals for (α-AHs + Cl)^−^ were recorded *via* ESMS with a digital stopwatch.

The mass spectrometer was operated under the following conditions: drying gas (N_2_) flow rate, 12 L min^−1^; drying gas temperature, 340 °C; inlet voltage, +3.5 kV relative to ground; fragmentor voltage, 60 V. All solutions were prepared in ultrapure water (resistivity ≥ 18.2 MΩ cm at 298 K) from a Millipore Milli-Q water purification system. α-Tp (95%, Tokyo Chemical Industry), ethanol (99.5%, Wako), 1-propanol (99.5%, Tokyo Chemical Industry), 2-propanol (99.5%, Tokyo Chemical Industry), HCl (37%, ACS reagent-grade, Sigma-Aldrich), and NaCl (99.999%, Sigma-Aldrich) were used as received.

### Theoretical calculations

Calculations for the Gibbs free energy barriers were performed with the Gaussian 16 program (revision C. 01).^27^ The relaxed scan of the potential energy surface between reactants and products was carried out with a step size of 0.5 Bohr to identify possible transition states by using the M06-2X functional^28^ and the 6-31G(d,p) basis set (*i.e.*, M06-2X/6-31G(d,p)).^29^ Geometrical optimization and frequency analysis of reactants, transition states and products were computed with the same method. The Gibbs free energy (*G*) and enthalpy (*H*) were calculated by eqn (1) and (2).

1 *G* = *E*^SP^ + *G*^corr^

2 *H* = *E*^SP^ + *H*^corr^

*G* ^corr^ and *H*^corr^ are thermal corrections of Gibbs free energy and enthalpy, which were estimated by M06-2X/6-31G(d,p), while *E*^SP^ is the single-point energy refined by using the M06-2X functional implemented with Grimme's third-generation empirical dispersion correction (D3)^30^ and the 6-311G(2d,p) basis set^29^ (*i.e.*, M06-2X-D3/6-311G(2d,p)). The SMD implicit solvation model was employed to simulate the water environment in all of the calculations.^31^ The temperatures for calculating the Gibbs free energy, enthalpy, and entropy for all the reactants, transition states, and products were set at 288, 298, 308, and 318 K. Pseudo-first-order kinetics was assumed for all the H^+^-catalyzed and water-catalyzed decomposition processes, and the total reaction rate coefficients (*k*) of α-AHs decay were evaluated by their linear combination (eqn (3)).3
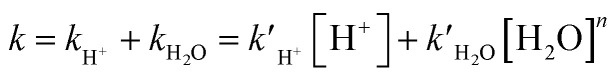
where [H^+^] is the concentration of H^+^ and is set as 3.16 × 10^−5^ M (corresponding to pH = 4.5) for all the calculations, [H_2_O] is the molar concentration of water molecules, which is 55.5 M at the standard condition, *n* is the number of water molecules involved in the unit reactions. 
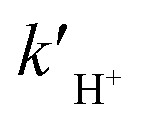
 and 
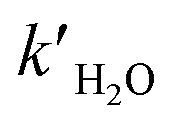
 are the reaction rate coefficients of α-AHs decomposition corresponding to H^+^- and water-catalyzation, which can be estimated by the Eyring equation^32^ (eqn (4)).4
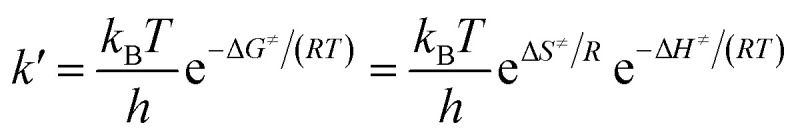
where Δ*G*^≠^, Δ*S*^≠^, and Δ*H*^≠^ are the activation changes of Gibbs free energy, entropy, and enthalpy, respectively. *k*_B_ is the Boltzmann constant; *h* is the Planck constant; *T* is the temperature; and *R* is the gas constant. For reactions with Gibbs free energy barriers but without transition states, Δ*G*^≠^, Δ*S*^≠^, and Δ*H*^≠^ were calculated by the difference of *G*, *S*, and *H* between products and reactants. The activation energy (*E*_a_) for reactions was estimated using eqn (5) according to the correlation between the Arrhenius equation and the transition state theory:

5 *E*_a_ = Δ*H*^≠^ + *RT*

## Results and discussion

### Decays of α-AHs in alcohol:water solutions at different temperatures

**Fig. 2:**
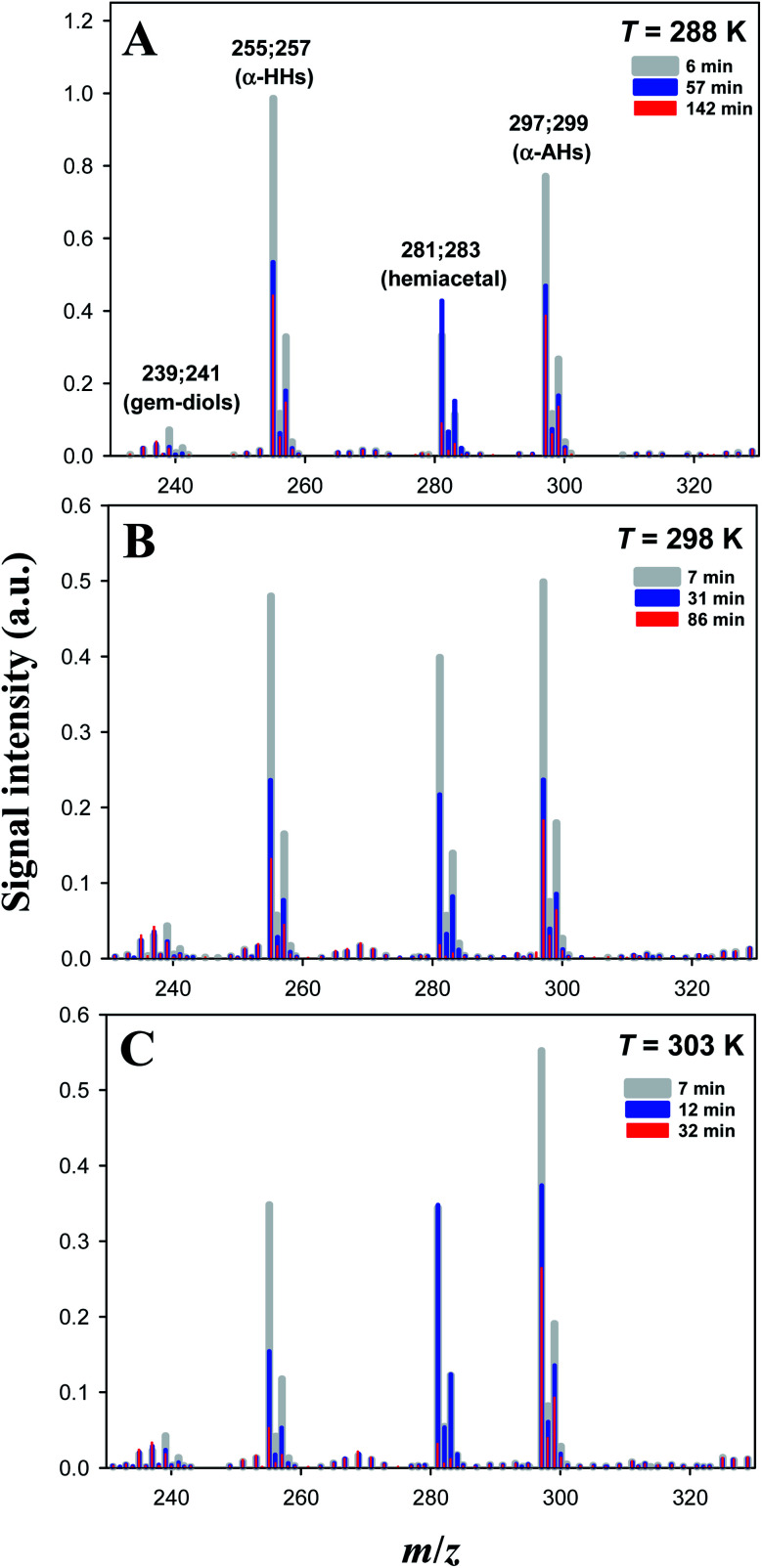
Temporal dependence of negative-ion mass spectra of mixtures obtained by ozonolysis ([O_3_]_0_ = 0.06 ± 0.01 mM) of solutions of α-terpineol (1 mM) and NaCl (0.2 mM) in a 1-propanol : water (1 : 1 = vol/vol) solution at *T* = 288 K (A), 298 K (B) or 303 K (C) acidified by 0.05 mM HCl at pH 4.5. Ion signals at *m*/*z* 239; 241, *m*/*z* 255; 257, *m*/*z* 281; 283, and *m*/*z* 297; 299 correspond to Cl^−^ adducts of C_10_ geminal diols, C_10_ α-HHs, C_13_ hemiacetals, and C_13_ α-AHs, respectively. The small ion signals at *m*/*z* 267; 269; 271; 273 correspond to Na_4_Cl_5_^−^ clusters. See our previous study^16^ for assignments using D_2_O and H_2_^18^O.

Time-dependent negative-ion mass spectra of solutions obtained by the reaction of O_3_(aq) + α-Tp/NaCl(aq) in 1-propanol : water (1 : 1 = vol/vol) at pH 4.5 adjusted by addition of HCl at *T* = 288, 298 and 303 K are shown in Fig. 2.

**Scheme 1:**
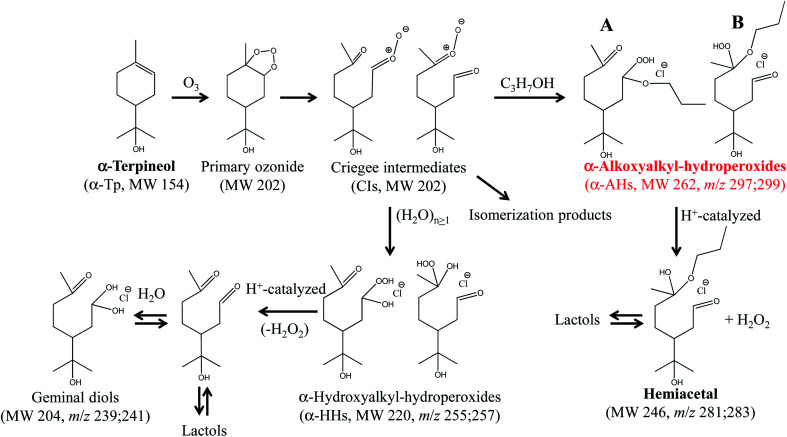
The formation and decomposition of α-AHs (A; secondary –OOH, B; tertiary –OOH) and α-HHs derived from ozonolysis of α-terpineol in 1-propanol : water (1 : 1=vol/vol) solutions. The likely isomers are shown.^16^

Ozonolysis of α-Tp begins with incorporation of O_3_ into the C=C double bond of α-Tp, resulting in the formation of a primary ozonide,^26,33^ which then isomerizes to CIs in the solution. Then, the CIs in the alcohol : water (1 : 1 = vol/vol) mixture isomerize,^25,34–36^ react with (H_2_O)_*n*≥1_ to produce α-HHs,^12,13,36^ or react with alcohol to produce α-AHs (Scheme 1). The doublet peaks at *m*/*z* 255; 257 and *m*/*z* 297; 299 in Fig. 2 were assigned to the C_10_ α-HH–Cl^−^ and C_13_ α-AH–Cl^−^ derived from α-Tp: 255; 257 = 154 (α-Tp) + 48 (O_3_) + 18 (H_2_O) + 35; 37 (Cl^−^), and 297; 299 = 154 (α-Tp) + 48 (O_3_) + 60 (1-propanol) + 35; 37 (Cl^−^).^12,16^ From the mass-specific and reaction time-dependent characterization of the products formed in 1-propanol:H_2_^18^O and 1-propanol:D_2_O solutions, we previously identified the α-HH-Cl^−^ and α-AH-Cl^−^ and corresponding decomposition products.^12,16^ We note that in the absence of HCl (pH 5.9), the *m*/*z* 297; 299 signals decreased to less than 20% of their maximum value and persisted even after several hours,^16^ implying the decomposition of α-AHs is H^+^-catalyzed. We previously found that the decay of the signal at *m*/*z* 297; 299 became faster as pH decreased from 5.9 to 3.8 in the solution at room temperature.^16^ The peaks at *m*/*z* 239; 241 were assigned to the C_10_ geminal diols, a decomposition product from C_10_ α-HHs. The doublet peaks at *m*/*z* 281; 283 in Fig. 2 were assigned to C_13_ hemiacetals, a decomposition product of C_13_ α-AHs; hemiacetals = 297; 299 (α-AH–Cl^−^) − 16 (O), where the –OOH of the α-AHs is replaced by the –OH from water (Scheme 1). Experiments for the reaction of O_3_(aq) + α-Tp/NaCl(aq) in aqueous mixtures with ethanol and 2-propanol confirmed the formation of C_12_ and C_13_ α-AHs, respectively. In our previous work, the α-AH signals appearing at *m*/*z* = 154 (α-Tp) + 48 (O_3_) + alcohol's MW + 35/37 (Cl^−^) and corresponding hemiacetal products (−16 Da) were also confirmed for methanol and 2-propanol-d_8_ in alcohol : water (1 : 1 = vol/vol) solutions.^16^ Therefore, it is evident that the α-Tp CIs react with the C_≤3_ alcohols to form the α-AHs, that decompose into corresponding hemiacetals in the liquid phase.

We previously showed, by using D_2_O and H_2_^18^O solvents, that the hemiacetals can be formed *via* an H^+^-catalyzed decomposition involving an H_2_O_2_ emission and an H_2_O addition; α-AH (+H^+^) − H_2_O_2_ + H_2_O (−H^+^).^16^ The hemiacetals are expected to further transform into MS-silent lactols (Scheme 1). We will discuss the decomposition mechanism in the theoretical calculation section.

**Fig. 3:**
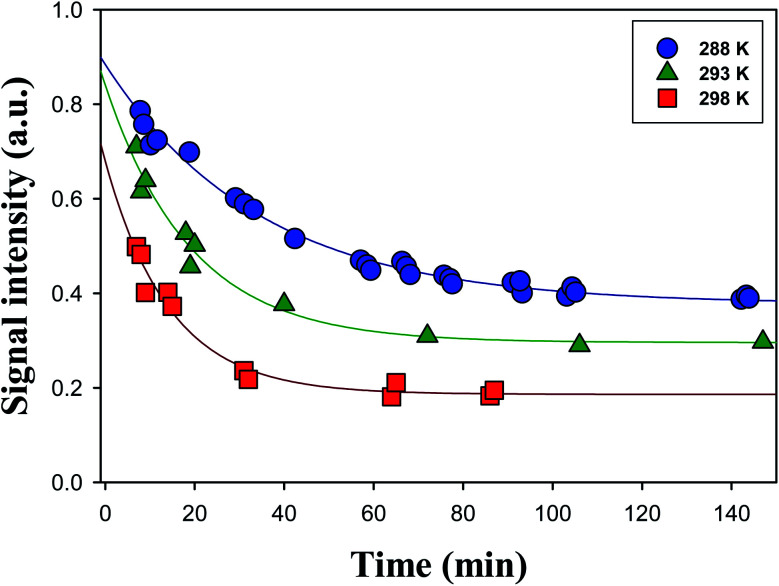
Decay profiles of the Cl^−^ adducts of the C_13_ α-AHs (*m*/*z* 297) generated by ozonolysis of α-Tp (1 mM α-terpineol, 0.2 mM NaCl) at [O_3_]_0_ = 0.06 mM in a 1-propanol : water (1 : 1 = vol/vol) solution at *T* = 288 K (circles), 293 K (triangles) or 298 K (squares) at pH 4.5 adjusted by 0.05 mM HCl. Lines indicate fittings of signal intensities (*S*) to single-exponential functions with baselines. See the text for details.

Fig. 3 shows time series of the profiles of the α-AH signals obtained for the reaction of O_3_(aq) + α-Tp/NaCl(aq) in 1-propanol : water (1 : 1 = vol/vol) at pH 4.5 adjusted by 0.05 mM HCl at *T* = 288 K, 293 K, and 298 K. The time profiles of α-AHs showed a single-exponential decay that did not go to zero under the conditions of the present study.

The decays of the *m*/*z* 297 signals were fitted with a single-exponential function with a baseline *S* = *S*_0_ + *A* exp(−*kt*). The decays of α-AHs derived from α-Tp CIs + 2-propanol and ethanol were also fitted with the single-exponential function with a baseline. As previously discussed,^16^ the baseline (*S*_0_) was attributed to inert isomers of the *m*/*z* 297; 299, such as the specific α-AHs R_1_C(–H)(–OOH)(–OR′) possessing a secondary –OOH (A in Scheme 1) and/or cyclic peroxyhemiacetals that could not decompose at the current timescales. It has been reported that tertiary hydroperoxides can produce corresponding tertiary alcohols under acidic conditions, due to the stabilization of the corresponding carbenium ion.^37^ In that case, the observed 1^st^ order decays and corresponding *k* values were attributed to α-AHs possessing a tertiary –OOH (B in Scheme 1). This interpretation is supported by the theoretical calculations as shown below. The observation that the larger *S*_0_ values were obtained at lower temperatures (Fig. 3) implies that the relative yield of α-AHs possessing a secondary *versus* tertiary –OOH (A/B in Scheme 1) increases as temperature decreases. The obtained *k* values for different temperatures are summarized in Table 1. The *k* values are means ± SDs for three independent experiments.

**Table 1:** Rate coefficients for decay of the C_13_ α-AHs derived from α-Tp ozonolysis in 1-propanol:water solutions at pH 4.5 at different temperatures^a^

Temperature (K)	*k*_pH 4.5_ (s^−1^)	*τ*_1/e_ (minutes)
288	(5.3 ± 0.2) × 10^−4^	31
293	(7.5 ± 1.4) × 10^−4^	22
298	(1.2 ± 0.3) × 10^−3^	14
303	(1.6 ± 0.7) × 10^−3^	10
308	(2.1 ± 1.4) × 10^−3^	8

a Experimental conditions: [α-Tp]_0_ = 1 mM, [NaCl]_0_ = 0.2 mM, [O_3_]_0_ = 0.06 mM, pH 4.5. The *k* values are means ± SDs for three independent experiments. *τ*_1/e_ = 1/*k*.

**Fig. 4:**
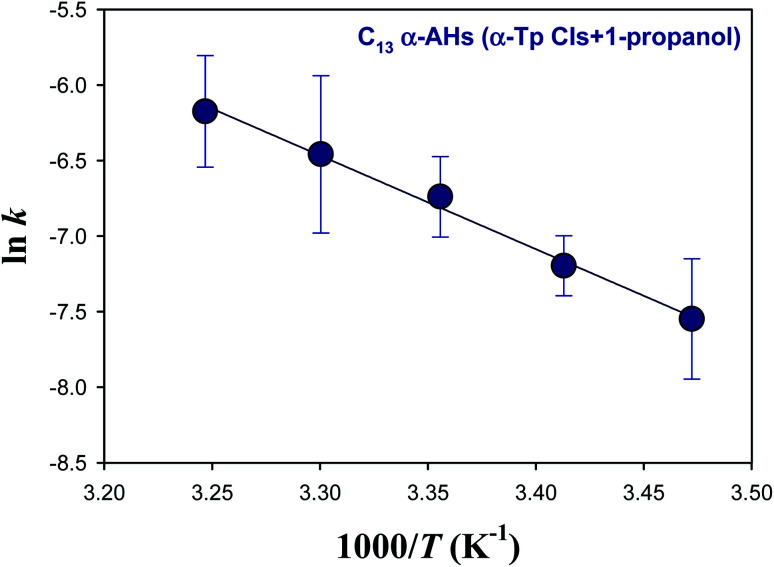
Arrhenius plot of the rate coefficients (*k*) for the decay of the C_13_ α-AHs generated by ozonolysis of aqueous α-terpineol in the presence of 1-propanol at pH 4.5.

Fig. 4 shows an Arrhenius plot (ln *k vs.* 1/*T*) for the decomposition of α-AHs derived from ozonolysis of α-Tp in 1-propanol:water solutions at pH 4.5. The linear regression yielded a preexponential factor (*A*) of 1.2 × 10^6^ s^−1^ (ln *A* = 14.0 ± 1.0) and an *E*_a_ value of 12.3 ± 0.6 kcal mol^−1^. The uncertainties were obtained from the standard errors of the intercept and slope, respectively. Temperature dependent *k* was expressed as *k* = 1.2 × 10^6^ exp[−(51.5 ± 2.5) × 10^3^/*RT*] s^−1^, where *R* is the gas constant.

The temporal profiles of α-AHs derived from α-Tp CIs + 1-propanol in solution at pH 4.0 (adjusted by adding 0.1 mM HCl) were measured to determine *k*. The obtained *k* and the Arrhenius plot at pH 4.0 are shown in Table S1 and Fig. S2.† The derived *E*_a_ for pH 4.0 was 14.4 ± 0.6 kcal mol^−1^, which is slightly (by 2.1 kcal mol^−1^) larger than that for pH 4.5.

We also obtained a series of the profiles of the α-AH signals for the reaction of α-Tp CIs with 2-propanol and ethanol at pH 4.5 at different temperatures. All the decays of α-AHs showed single-exponential functions *S* = *S*_0_ + *A* exp(−*kt*), consistent with the case of α-AHs derived from CIs + 1-propanol. Derived *k* values were summarized in Tables 2 and 3.

**Table 2:** Rate coefficients for decay of C_13_ α-AHs derived from α-Tp ozonolysis in 2-propanol:water solutions at pH 4.5 at different temperatures^a^

Temperature (K)	*k*_pH 4.5_ (s^−1^)	*τ*_1/e_ (minutes)
293	(4.0 ± 0.5) × 10^−4^	42
298	(7.0 ± 1.4) × 10^−4^	24
303	(1.1 ± 0.2) × 10^−3^	15
308	(1.9 ± 0.4) × 10^−3^	9

a Experimental conditions: [α-Tp]_0_ = 1 mM, [NaCl]_0_ = 0.2 mM, [O_3_]_0_ = 0.06 mM, pH 4.5. The *k* values are means ± SDs for three independent experiments. *τ*_1/e_ = 1/*k*.

**Table 3:** Rate coefficients for decay of C_12_ α-AHs derived from α-Tp ozonolysis in ethanol:water solutions at pH 4.5 at different temperatures^a^

Temperature (K)	*k*_pH 4.5_ (s^−1^)	*τ*_1/e_ (minutes)
288	(7.2 ± 0.5) × 10^−4^	23
293	(1.1 ± 0.2) × 10^−3^	15
298	(1.8 ± 0.2) × 10^−3^	9
303	(2.3 ± 0.3) × 10^−3^	7

a Experimental conditions: [α-Tp]_0_ = 1 mM, [NaCl]_0_ = 0.2 mM, [O_3_]_0_ = 0.06 mM, pH 4.5. The *k* values are means ± SDs for three independent experiments. *τ*_1/e_ = 1/*k*.

**Fig. 5:**
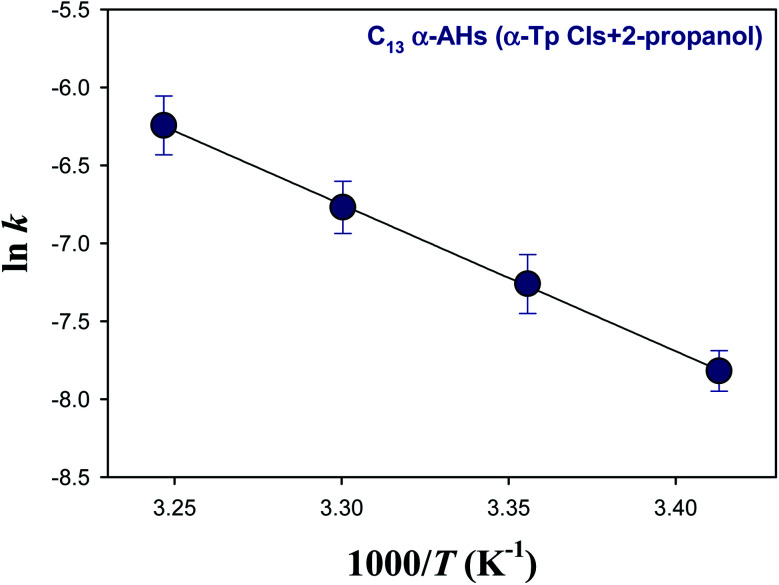
Arrhenius plot of the rate coefficients (*k*) for the decay of the C_13_ α-AHs generated by ozonolysis of aqueous α-terpineol in the presence of 2-propanol at pH 4.5.

**Fig. 6:**
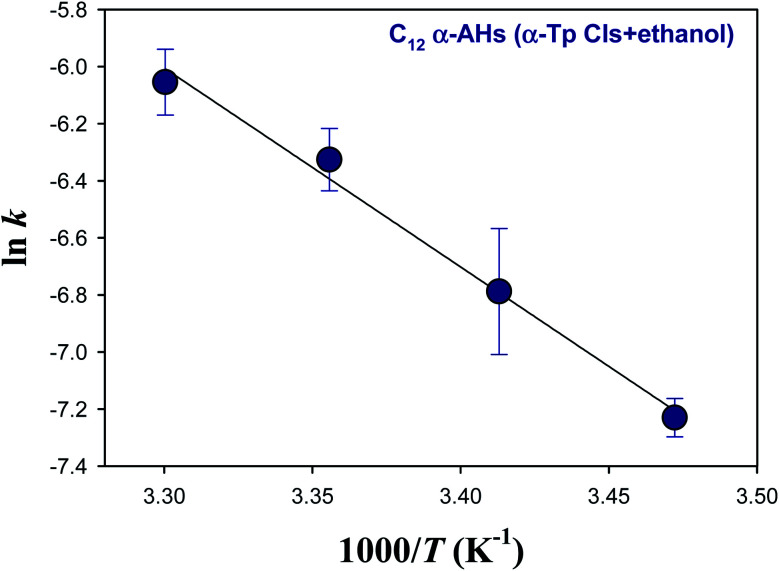
Arrhenius plot of the rate coefficients (*k*) for the decay of the C_12_ α-AHs generated by ozonolysis of aqueous α-terpineol in the presence of ethanol at pH 4.5.

Fig. 5 and 6 show Arrhenius plots for the decomposition of α-AHs derived from α-Tp CIs + 2-propanol and α-Tp CIs + ethanol at pH 4.5, which provide *E*_a_ of 18.7 ± 0.3 and 13.8 ± 0.9 kcal mol^−1^, respectively.

### Theoretical calculations for the decay of α-AHs in the aqueous phase

**Fig. 7:**
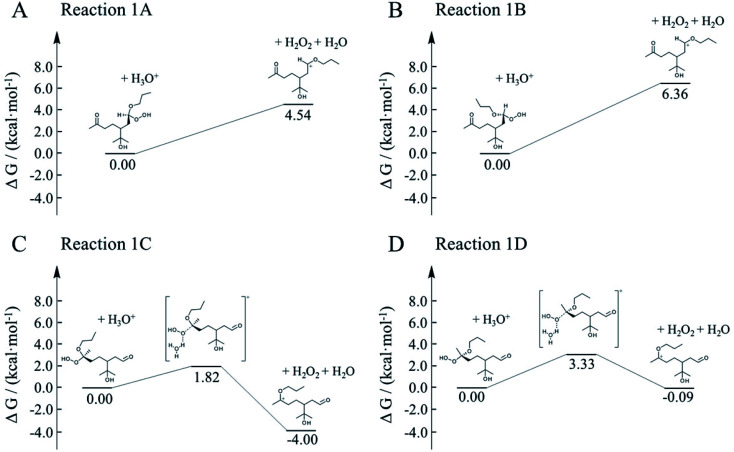
Gibbs free energy profiles of H^+^-catalyzed decomposition of C_13_ α-AHs (α-terpineol CIs + 1-propanol).

**Fig. 8:**
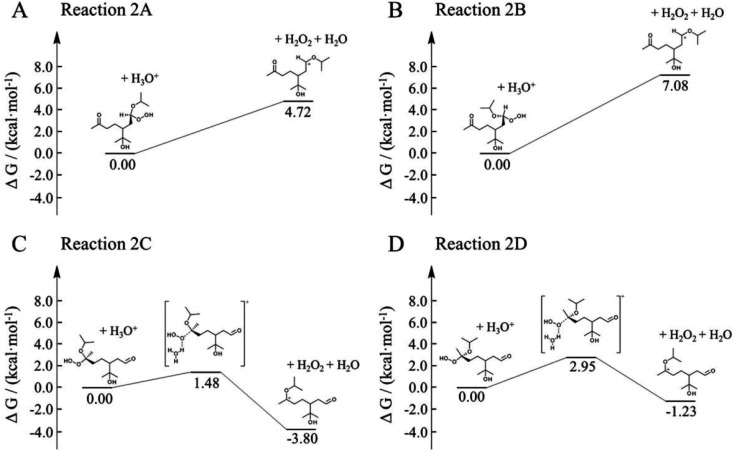
Gibbs free energy profiles of H^+^-catalyzed decomposition of C_13_ α-AHs (α-terpineol CIs + 2-propanol).

Here, we employed first-principle calculations to estimate the Gibbs free energy barriers of H^+^-catalyzed decomposition of α-AHs (Fig. 7 and 8) and the subsequent formation of hemiacetals (Fig. S3 and S4†). The Cartesian coordinates for the structural geometry used in the calculations are summarized in Table S2.† Since α-Tp potentially produces two kinds of CIs (Scheme 1) and each CI leads to two chiral isomers of α-AHs in the reaction with alcohols, the detected formula of each α-AH may have four possible structures (A, B, C, D in Fig. 7 and 8).

All the Gibbs free energy barriers in Fig. 7 and 8 exhibit low values (<10 kcal mol^−1^), supporting the high feasibility of H^+^-catalyzed decomposition mechanisms. The Gibbs free energy profiles of the subsequent hemiacetal formation also show small barriers (<4 kcal mol^−1^) or even no barriers (Fig. S3 and S4†), supporting the rapid hydration of carbenium ions. Since water molecules are ubiquitous in the aqueous environment, the hemiacetal formation is expected to be much faster than the carbenium ion formation. Hence, the H^+^-catalyzed decomposition of α-AH to carbenium ions should be the rate-determining steps of our proposed H^+^-catalyzed mechanisms of α-AH decay, and the reactions in Fig. 7 and 8 can be used to determine the *E*_a_ and the pseudo-first-order reaction rate coefficients at pH 4.5 (Table 4). However, the theoretical *E*_a_ values are about 4–15 kcal mol^−1^ lower than the apparent activation energy measured by experiments, while the calculated reaction rate coefficients are 6–11 orders of magnitude larger than the experimental results. This implies that other pathways of H^+^-catalyzation with tight concerted transition states may exist and lead to slower α-AH decomposition observed in our experiments. As the α-AHs are surrounded by ubiquitous water molecules in the solution, it is possible that water molecules participate in the H^+^-catalyzed channels and decelerate the α-AH decay by increasing the energy barriers. However, the exact reaction rate with explicit participation of water molecules is hard to estimate owing to the highly flexible conformation of liquid water, which may generate a variety of transition states and hence require further studies.

**Table 4:** Theoretical activation changes of entropy (Δ*S*^≠^), activation energies (*E*_a_) and pseudo-first-order reaction rate coefficients at pH 4.5 (*k*_H^+^_) for the eight reactions in Fig. 7 and 8

Reaction	Δ*S*^≠^ (kcal mol^−1^ K^−1^)	*E*_a_ (kcal mol^−1^)				*k*_H^+^_ (s^−1^)			
		288 K	298 K	308 K	318 K	288 K	298 K	308 K	318 K
1A	0.013	8.9	8.9	9.0	9.0	1.4 × 10^6^	1.6 × 10^6^	1.9 × 10^6^	2.1 × 10^6^
1B	0.009	7.1	7.2	7.2	7.2	1.8 × 10^5^	2.3 × 10^5^	2.9 × 10^5^	3.6 × 10^5^
1C	0.004	3.7	3.7	3.8	3.8	2.6 × 10^7^	2.9 × 10^7^	3.1 × 10^7^	3.3 × 10^7^
1D	0.005	5.4	5.4	5.5	5.5	5.0 × 10^6^	5.7 × 10^6^	6.5 × 10^6^	7.3 × 10^6^
2A	0.011	8.5	8.6	8.6	8.7	1.1 × 10^6^	1.3 × 10^6^	1.5 × 10^6^	1.8 × 10^6^
2B	0.009	5.8	5.8	5.8	5.8	7.9 × 10^4^	1.1 × 10^5^	1.5 × 10^5^	1.9 × 10^5^
2C	0.005	3.4	3.5	3.5	3.5	3.8 × 10^7^	4.1 × 10^7^	4.4 × 10^7^	4.6 × 10^7^
2D	0.006	5.3	5.3	5.4	5.4	7.6 × 10^6^	8.6 × 10^6^	9.7 × 10^6^	1.1 × 10^7^

**Fig. 9:**
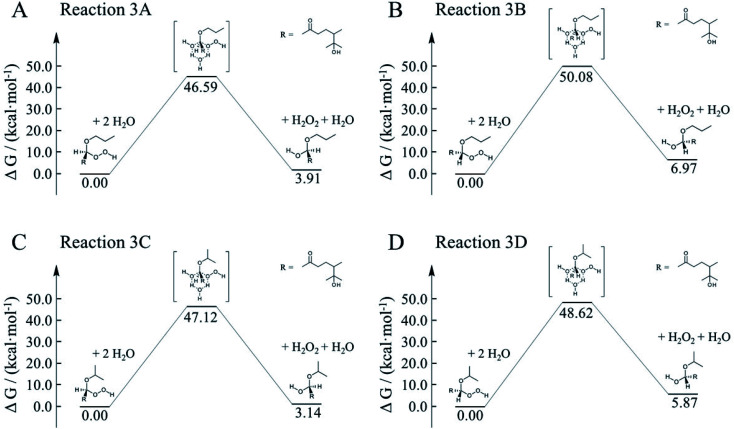
Gibbs free energy profiles of water-catalyzed decomposition of C_13_ α-AHs from α-terpineol CIs + 1-propanol (A and B) and α-terpineol CIs + 2-propanol (C and D).

To better constrain the influence of water participation in α-AH decay, kinetics of water catalyzation is further explored. Since the number of water molecules is much larger than H^+^, it is necessary to compare the water-catalyzed kinetics with those of the H^+^-catalyzed channels. Fig. 9 shows four possible pathways for the water-catalyzed reactions, where two water molecules (*i.e.*, *n* = 2 in eqn (3)) are assumed to be involved according to our previous study.^14^ Here we only present the mechanisms at the aldehyde group since the ketone group has a steric hindrance on the formation of the six-membered transitions states. All the Gibbs free energy barriers exhibit high values (>45 kcal mol^−1^), and all the activation changes of entropy are negative values, that are in sharp contrast with those in the H^+^-catalyzed reactions (Table 4). The theoretical *E*_a_ values are about 15–24 kcal mol^−1^ higher than the apparent activation energy measured by experiments, while the corresponding reaction rate coefficients are negligibly small compared with the measured values (Table 5). These results suggest that participation of water molecules may attribute to higher energy barriers in α-AH decomposition. In addition, since H^+^-catalyzation leads to much faster kinetics than water-catalyzation, the total reaction rate constants estimated by eqn (3) represent those of H^+^-catalyzation (Table 4). To further assess the H_2_O-catalyzed reactions under realistic experimental conditions, we may need to include more than three water molecules (*n* ≥ 3) or use a different theoretical approach. We also note that the presence of alcohol (*e.g.*, 1-propanol at 6.7 M) could modulate the activation energies of the α-AHs decomposition in alcohol:water mixtures.

**Table 5:** Theoretical activation changes of entropy (Δ*S*^≠^), activation energies (*E*_a_) and pseudo-first-order reaction rate coefficients (*k*_H_2_O_) for the four reactions in Fig. 9

Reaction	Δ*S*^≠^ (kcal mol^−1^ K^−1^)	*E*_a_ (kcal mol^−1^)				*k*_H_2_O_ (s^−1^)			
		288 K	298 K	308 K	318 K	288 K	298 K	308 K	318 K
3A	−0.039	35.5	35.5	35.5	35.6	8.4 × 10^−20^	1.3 × 10^−18^	1.7 × 10^−17^	1.8 × 10^−16^
3B	−0.047	36.6	36.6	36.7	36.7	1.9 × 10^−22^	3.6 × 10^−21^	5.6 × 10^−20^	7.3 × 10^−19^
3C	−0.047	33.7	33.7	33.8	33.8	3.3 × 10^−20^	5.3 × 10^−19^	7.2 × 10^−18^	8.4 × 10^−17^
3D	−0.048	34.7	34.8	34.8	34.8	2.4 × 10^−21^	4.2 × 10^−20^	6.2 × 10^−19^	7.7 × 10^−18^

Our theoretical calculations revealed that the reactions of α-AHs R_1_C(–H)(–OOH)(–OR′) possessing a secondary –OOH (*e.g.*, A in Scheme 1) leading to the formation of corresponding carbenium ions are energetically less favorable compared to α-AHs R_1_C(–R_2_)(–OOH)(–OR′) possessing a tertiary –OOH (*e.g.*, B in Scheme 1). The present calculations also confirmed that H_2_O_2_ could be an important byproduct of the decomposition of α-AHs in the aqueous phase, consistent with the previous theoretical result for α-Tp α-HHs.^15^ A previous iodometry analysis of a mixture of 1 mM α-Tp + 0.2 mM NaCl + 0.06 mM O_3_ + 0.1 mM HCl revealed that approximately 70% of O_3_ was converted into H_2_O_2_ and other peroxides in water.^38^ Another experimental study on the aqueous-phase decomposition of α-acyloxyalkyl-hydroperoxides derived from α-pinene CIs with carboxylic acids found that H_2_O_2_ is a key decomposition product.^39^

### Comparison of the activation energies for the decomposition of α-AHs and related ROOHs in aqueous solutions

The experimentally determined *E*_a_ values for the decomposition of α-AHs and related ROOHs in liquid phases are summarized in Table 6. The decay rate coefficients for α-AHs, α-HHs, and α-acyloxyalkyl-hydroperoxides at pH ∼ 4.5 listed in the Table 6 are in the orders of 10^−4^ to 10^−3^ s^−1^.

**Table 6:** Activation energies for decay of the α-AHs and related ROOHs in liquid phases

ROOH	*E*_a_ (kcal mol^−1^)
**C**_**13**_ **α-AHs (α-terpineol CIs + 1-propanol) at pH 4.5**	**12.3 ± 0.6**
**At pH 4.0**	**14.4 ± 0.6**
**C**_**13**_ **α-AHs (α-terpineol CIs + 2-propanol) at pH 4.5**	**18.7 ± 0.3**
**C**_**12**_ **α-AHs (α-terpineol CIs + ethanol) at pH 4.5**	**13.8 ± 0.9**
C_16_ α-acyloxyalkyl hydroperoxides (α-pinene CIs + adipic acid) at pH 4.4^a^	15.0 ± 1.0
C_20_ α-acyloxyalkyl hydroperoxides (α-pinene CIs + pinonic acid) at pH 4.4^a^	14.5 ± 1.6
C_10_ α-terpineol α-HHs at pH 4.5^b^	19.2 ± 0.5
C_10_ terpinen-4-ol α-HHs at pH 6.2^b^	17.1 ± 0.2
C_1_ hydroxymethyl hydroperoxide at pH 7.1^c^	22.9

a Ref. 39.

b Ref. 15.

c Ref. 40.

The *E*_a_ values for the decomposition of the α-AHs derived from α-Tp CIs + alcohols examined in the present study range in 12–15 kcal mol^−1^, except for α-AHs from 2-propanol (18.7 ± 0.3 kcal mol^−1^). It can be argued that a steric hindrance of isopropyl group would limit the accessibility of (H_2_O)_*n*_H^+^ to the –OOH moiety, resulting in a larger *E*_a_. However, our theoretical calculations show that the *n*-propyl and isopropyl groups have little influence on the activation energy, revealing that the accessibility of (H_2_O)_*n*_H^+^ to the –OOH group is not hindered. This finding suggests that other mechanisms may also play a role in α-AH decomposition.

It was reported that the decomposition of *tert*-alkyl-hydroperoxides R_1_R_2_R_3_C–OOH yields the corresponding *tert*-alkyl-alcohols R_1_R_2_R_3_C–OH under acidic conditions, due to the high stabilization of corresponding carbenium ions.^37,41^ The assumed mechanism involves a protonation of the O-atom next to the C-atom of R_1_R_2_R_3_C–OOH, releasing H_2_O_2_ by leaving a positive charge on the C-atom. In that case, the obtained *E*_a_ values were attributed to specific α-AHs possessing a tertiary –OOH (*e.g.*, B in Scheme 1). The formed carbenium ion is rapidly hydrated to form an oxocarbenium ion, which releases an H^+^ to form the corresponding alcohol, as supported by the low Gibbs free energy barriers (0–4 kcal mol^−1^) in our calculations (Fig. S3 and S4†).

### Atmospheric implications

ROOHs possessing single or multiple –OOH moieties are a class of ubiquitous intermediate species that are formed by the oxidation of VOCs. The OH oxidation of VOCs produces RO_2_ radicals, which then undergo bimolecular reactions or intramolecular H-atom abstractions and O_2_ addition to form HOM, whereas the ozonolysis of VOCs with C=C double bond(s) produces CIs, which in most cases end up forming ROOHs with multifunctionalities. The important point is that both OH and O_3_ oxidation of VOCs can result in the production of ROOHs, although the quantitative yields of ROOHs *via* such processes are not known yet. Because of the low volatilities and high hydrophilicities, ROOHs are readily taken up into atmospheric condensed phases such as aerosols, fog/cloud droplets, and wet films of plants and soils. The kinetic data of ROOHs in atmospheric condensed phases is a key parameter in atmospheric modeling. In this article, we determined the temperature-dependent decomposition rate coefficients and the activation energies of α-AHs [R_1_R_2_C(–OOH)(–OR′)], a class of important ROOHs generated from the reaction of CIs with alcohols, in the liquid phase. We found longer lifetimes for the α-AHs in solutions at lower temperatures. For example, the lifetime of C_13_ α-AHs derived from α-Tp CIs + 1-propanol is changed from 8 min at 308 K to 31 min at *T* = 288 K. This result suggests that the lifetimes of α-AHs in aerosols are affected not only by the pH of reaction media but also by local temperatures under different environmental conditions such as altitude, day/night, season, and weather. Another implication is that, as in the case of α-HHs and other ROOHs, the storage of aerosol samples at lower temperatures would be a promising way to extend the lifetime of α-AHs for analysis in field measurements and chamber experiments.^42^ The derived *E*_a_ values for the decomposition of the α-AHs of the α-Tp CIs + alcohols would be useful for atmospheric modeling as a representative value of ROOH possessing –OOH and –OR′.

Both our experiments and theoretical calculations revealed that the H^+^-catalyzed conversion of ROOH into ROH, which is accompanied by the formation of H_2_O_2_, may be a general mechanism that produces H_2_O_2_ and multifunctionalized species in atmospheric condensed phases^3,43,44^ and contributes to the oxidative potential and toxicity of secondary organic aerosols.^45^ The rate of the proposed H^+^-catalyzed decomposition of α-AHs in ambient aerosol particles could be faster than the rate *via* photolysis by solar radiation or decomposition catalyzed by transition metals. We recently found that the reaction of α-Tp α-HHs with Fe^2+^ is outcompeted by H^+^-catalyzed decomposition of α-HHs at ambient concentrations of Fe^2+^ and H^+^ found in atmospheric condensed phases.^38^ Further work to test the effects of structures and functionalities on the decomposition of ROOHs in the aqueous phase is underway. Our theoretical calculations imply the existence of different reaction pathways, such as water-catalyzed reactions, that could be correlated with the fates of α-AHs in ambient particles under variable relative humidity.

## Conclusion

We report an experimental study of the liquid-phase decompositions of C_12_ or C_13_ α-alkoxyalkyl-hydroperoxides (α-AHs) derived from ozonolysis of α-terpineol in the presence of 1-propanol, 2-propanol, and ethanol as a function of temperature. The temporal profiles of α-AH signals, detected as chloride-adducts by negative ion electrospray mass spectrometry, showed single-exponential decay, and the derived first-order rate coefficient *k* for α-AH decomposition increased as temperature increased, *e.g.*, *k*(288 K) = (5.3 ± 0.2) × 10^−4^, *k*(298 K) = (1.2 ± 0.3) × 10^−3^, *k*(308 K) = (2.1 ± 1.4) × 10^−3^ s^−1^ for C_13_ α-AHs derived from the reaction of α-terpineol Criegee intermediates with 1-propanol in solution at pH 4.5. Arrhenius plot analysis yielded activation energy (*E*_a_) of 12.3 ± 0.6, 18.7 ± 0.3 and 13.8 ± 0.9 kcal mol^−1^ for the decomposition of α-AHs derived from the reaction of α-Tp CIs with 1-propanol, 2-propanol and ethanol, respectively. Theoretical kinetic and thermodynamic calculations revealed that H^+^-catalyzed mechanism plays an important role in the decomposition of these α-AHs in acidic water, while water molecules may also participate in the H^+^-catalyzed mechanism and influence the kinetics. The rate of the proposed H^+^-catalyzed decomposition of α-AHs in ambient aerosol particles could be faster than that of other degradation processes. The proposed decomposition of α-AHs could act as a source of H_2_O_2_ and multifunctionalized species in atmospheric condensed phases.

## Author contributions

S. E. designed the research; M. H. and S. E. performed the experiments; K. C. performed the theoretical calculations; S. E. wrote the first draft of the manuscript; and all of the authors analyzed the data and contributed to revising the manuscript.

## Conflicts of interest

There are no conflicts to declare.

## Supplementary Material

EA-002-D1EA00076D-s001
