# Supplementary material for: Decomposition mechanism of α-alkoxyalkyl-hydroperoxides in the liquid phase: temperature dependent kinetics and theoretical calculations
Source: Environ Sci Atmos. 2022 Jan 17;2(2):241–51. doi: 10.1039/d1ea00076d (PMC8929293; doi:10.1039/d1ea00076d)
Supplement: EA-002-D1EA00076D-s001 [file EA-002-D1EA00076D-s001.pdf]

Electronic Supplementary Material (ESI) for Environmental Science: Atmospheres.
This journal is © The Royal Society of Chemistry 2022

Supplementary Information for

Decomposition mechanism of α-alkoxyalkyl-hydroperoxides in the liquid phase: Temperature dependent kinetics and theoretical calculations

Mingxi Hu[a], Kunpeng Chen[b], Junting Qiu[a], Ying-Hsuan Lin[b], Kenichi Tonokura[a], and Shinichi Enami*[c]

[a]Graduate School of Frontier Sciences, The University of Tokyo, 5-1-5 Kashiwanoha, Kashiwa 277-8563, Japan

[b]Department of Environmental Sciences, University of California, Riverside, California 92521, United States

[c]National Institute for Environmental Studies, 16-2 Onogawa, Tsukuba 305-8506, Japan.

E-mail: enami.shinichi@nies.go.jp

No. of Supporting Pages: 77

No. of Supporting Tables: 2

No. of Supporting Figures: 4

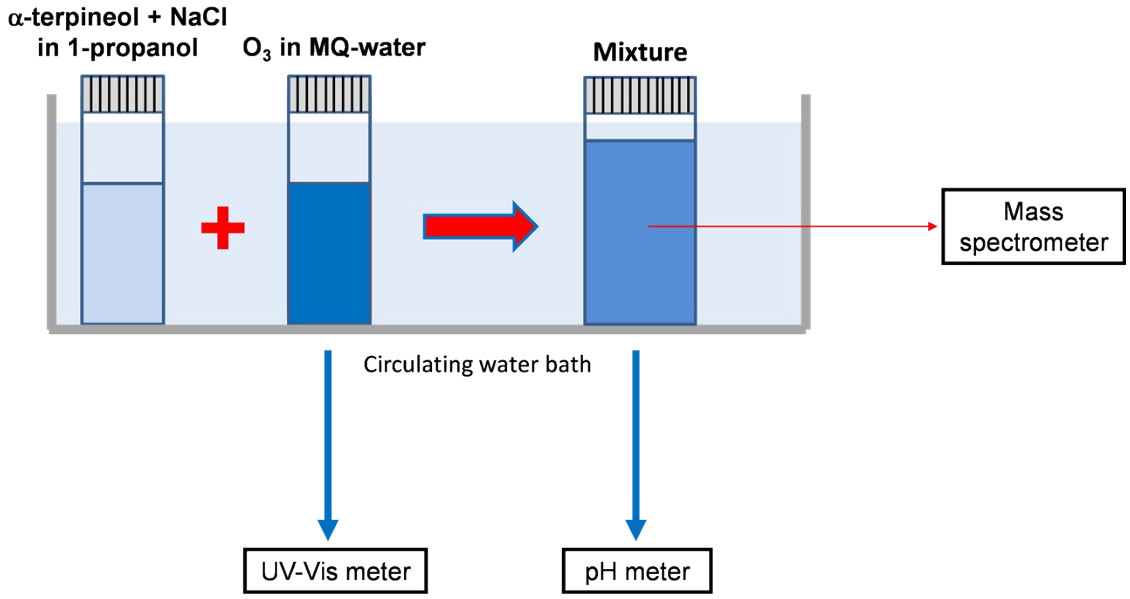

Figure S1 – Schematic procedure of present experiment.

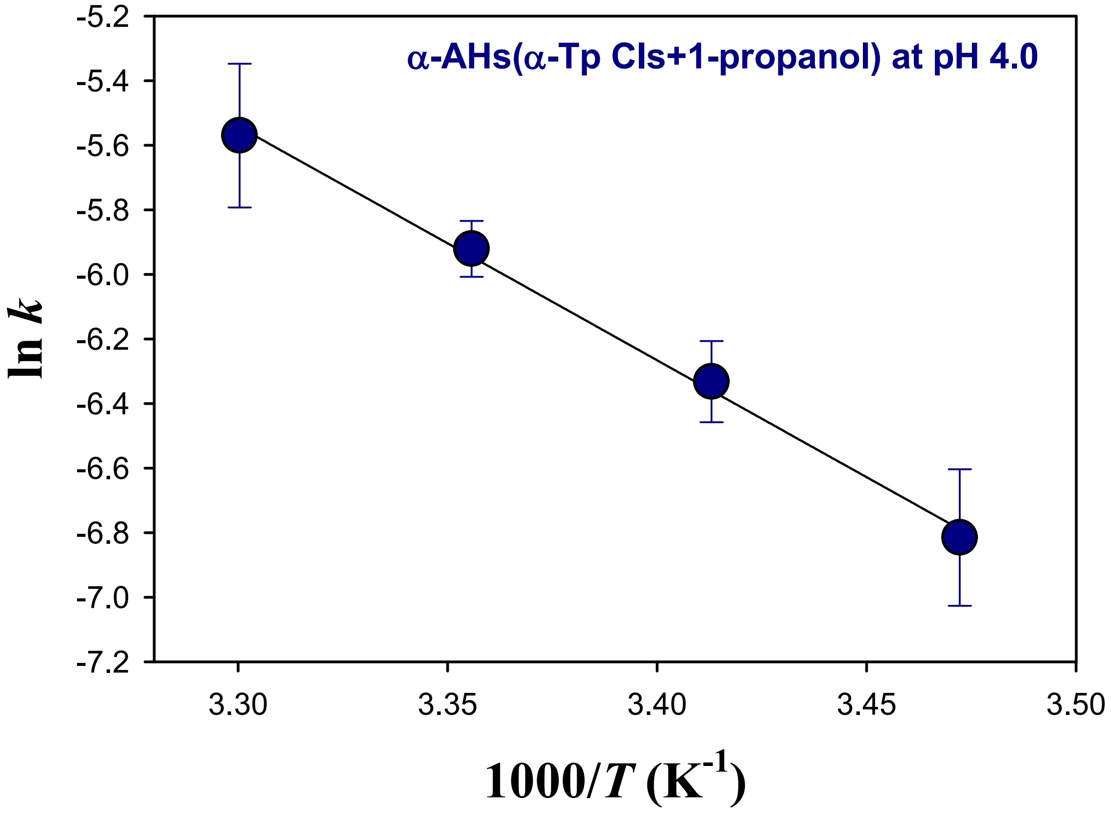

Figure S2 –Arrhenius plot of the rate coefficients (*k*) for the decay of the α-alkoxyalkyl-hydroperoxides (α-AHs) generated by ozonolysis of α-terpineol in 1-propanol:water (1:1 = vol:vol) at pH 4.0.

Figure S3 – Gibbs free energy profiles of hemiacetal formation from the carbenium ions produced by H^+-catalyzed decomposition of C_{13} α-AHs (α-terpineol CIs + 1-propanol).

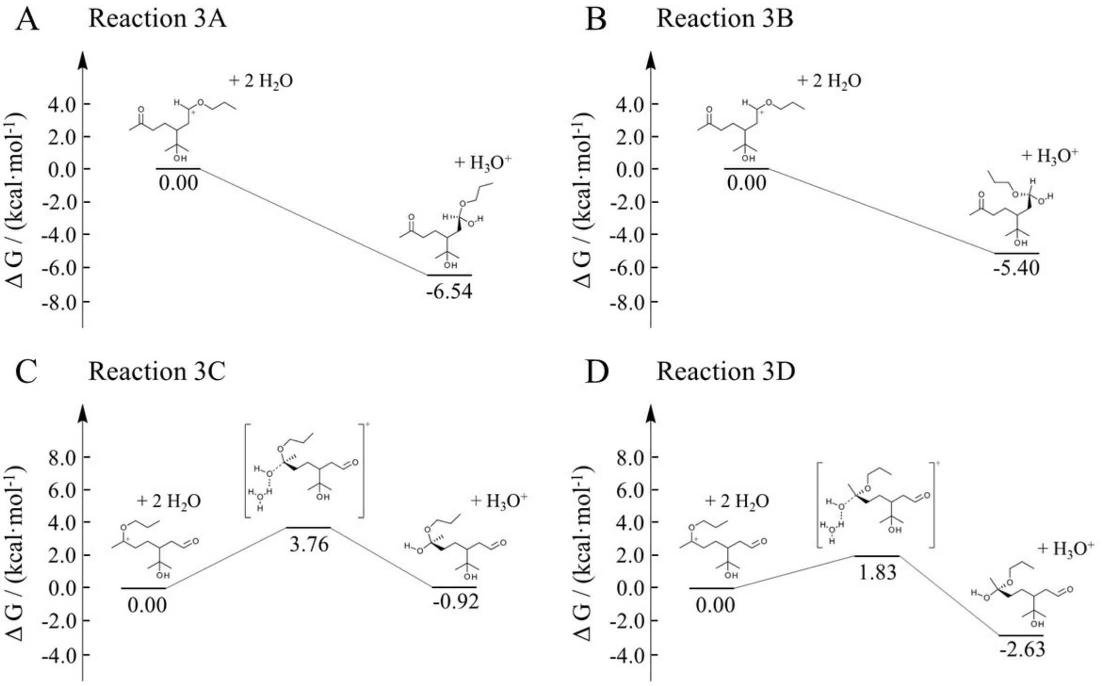

Figure S4 – Gibbs free energy profiles of hemiacetal formation from the carbenium ions produced by H^+-catalyzed decomposition of C_{13} α-AHs (α-terpineol CIs + 2-propanol).

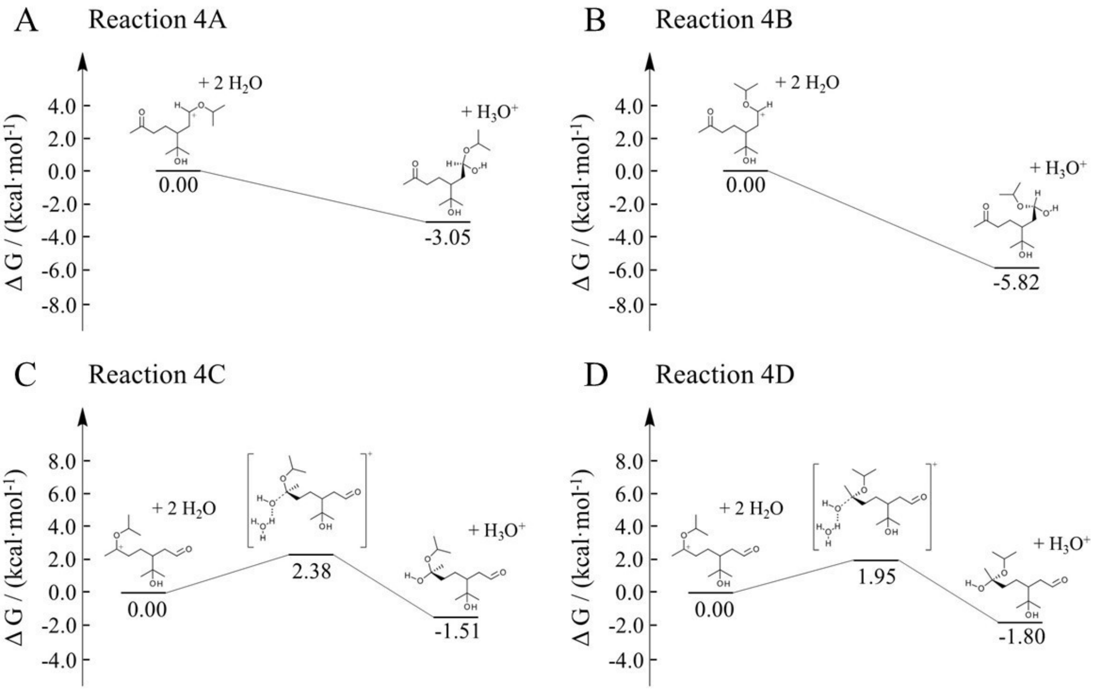

Table S1. Rate coefficients and lifetimes for decay of the α-AHs derived from α-terpineol ozonolysis in 1-propanol:water solutions at pH 4.0 adjusted by 0.1 mM HCl. $\tau_{1/e} = 1/k$.

Temperature (K)	$k_{pH4.0}$ (s^{-1})	$\tau_{1/e}$ (minutes)
288	$(1.1 \pm 0.2) \times 10^{-3}$	15
293	$(1.8 \pm 0.2) \times 10^{-3}$	9
298	$(2.7 \pm 0.2) \times 10^{-3}$	6
303	$(3.8 \pm 0.9) \times 10^{-3}$	4

Table S2. Cartesian coordinates for structures in the theoretical calculations

Reactant of Reaction 1A

C	0.66118300	-1.07661900	0.48222100
H	-0.12959500	-1.52152100	1.09321100
O	1.43992100	-0.28958600	1.42276300
O	1.40035800	-2.14050500	-0.01313900
O	1.78854100	-1.11484000	2.52485900
H	1.12450400	-0.84864900	3.18547700
C	0.10433400	-0.09116500	-0.52761700
H	0.92584000	0.43413200	-1.01928800
H	-0.38685800	-0.68474400	-1.30610400
C	-0.89725000	0.90155900	0.10758100
H	-0.80705700	0.84451100	1.20126600
C	-2.34037700	0.52901000	-0.25808300
H	-3.03594200	1.22844600	0.21307500
H	-2.47809700	0.62377100	-1.34194000
C	-0.54121500	2.35841800	-0.26662900
O	-0.49880800	2.39921500	-1.69921400
H	-0.25945000	3.30143700	-1.95366800
C	-2.72449100	-0.87695300	0.18630500
H	-2.11625800	-1.65275400	-0.29717000
H	-2.56157700	-1.00669500	1.26543700
C	-4.16955000	-1.22089500	-0.09093500
O	-4.93038300	-0.42327100	-0.61670500

C	-4.62390400	-2.59819100	0.31042600
H	-4.04780100	-3.34678900	-0.24215500
H	-4.42623500	-2.75839600	1.37433100
H	-5.68690900	-2.72281200	0.10406600
C	-1.58690400	3.35429500	0.23637300
H	-2.53649400	3.22798600	-0.28746000
H	-1.75817400	3.23652500	1.31082400
H	-1.23526100	4.37539100	0.05698300
C	0.82722700	2.74855200	0.29745600
H	0.82473900	2.70160600	1.39087900
H	1.62064100	2.09726500	-0.07914400
H	1.06649700	3.77481700	0.00005600
O	3.51750100	1.04944400	1.57550100
H	4.22737500	0.39668000	1.72946300
H	3.77470900	1.59064400	0.80490200
H	2.63618300	0.52781300	1.35956500
C	2.22623200	-1.94500000	-1.16837900
H	2.57733700	-2.95145900	-1.41280400
H	1.61936700	-1.59248800	-2.01050100
C	3.41906800	-1.02866500	-0.94641200
H	3.07736300	0.00693400	-0.83288200
H	3.91834000	-1.31496700	-0.01289200
C	4.38569000	-1.11838300	-2.12334800
H	3.88544900	-0.85202900	-3.05971500
H	5.23157500	-0.44008200	-1.99037800
H	4.77848900	-2.13399800	-2.22970000

Product of Reaction 1A

C	0.41463300	-1.26322300	0.25511800
H	-0.15279000	-1.50664200	1.15195400
O	1.57321500	-0.04977400	1.74912200
O	1.25463300	-2.16095800	-0.04679900
O	2.25916700	-1.12072100	2.38880100
H	2.11022400	-0.92162000	3.32734100
C	0.01905100	-0.13772500	-0.61648000
H	0.91356000	0.33193100	-1.03153300

H	-0.48507400	-0.60397400	-1.47862900
C	-0.91337300	0.87139200	0.08513200
H	-0.74775300	0.80468000	1.16836300
C	-2.38580800	0.54465400	-0.19694100
H	-3.02427900	1.24270100	0.35018200
H	-2.59134300	0.69205900	-1.26388200
C	-0.53116200	2.31304000	-0.32255000
O	-0.53812500	2.32672800	-1.75528000
H	-0.28371500	3.21708400	-2.03585300
C	-2.78493200	-0.86807500	0.21065500
H	-2.25167500	-1.63940100	-0.36171100
H	-2.54552200	-1.06115900	1.26553700
C	-4.25967900	-1.14626300	0.02485500
O	-5.01374100	-0.31226400	-0.45060300
C	-4.74960700	-2.50131500	0.45704100
H	-4.17663900	-3.28297500	-0.05010200
H	-4.58119300	-2.62155600	1.53168700
H	-5.81098300	-2.60995700	0.23409000
C	-1.53402500	3.33979300	0.20302600
H	-2.50510000	3.22686700	-0.28305700
H	-1.66606000	3.24304500	1.28496100
H	-1.16458700	4.34844300	-0.00729800
C	0.86570600	2.67238900	0.18731700
H	0.89887200	2.64640400	1.28113300
H	1.62654700	1.98927600	-0.20049900
H	1.12615400	3.68396400	-0.14035900
O	3.76670300	1.33246700	1.36296300
H	4.41853200	0.64943300	1.14957700
H	3.75341500	1.90348500	0.58227900
H	2.34190300	0.54025700	1.48950400
C	2.13099500	-2.04850300	-1.21739500
H	2.37830500	-3.08494200	-1.44429800
H	1.56187700	-1.61869000	-2.04339400
C	3.36095800	-1.24073400	-0.85638000
H	3.06811400	-0.21981000	-0.59058400
H	3.82849900	-1.68835000	0.02658400

C	4.33359500	-1.22382300	-2.03166200
H	3.87348600	-0.77202300	-2.91510800
H	5.22431000	-0.64447700	-1.77969500
H	4.64998500	-2.23751800	-2.29324000

Reactant of Reaction 1B

C	1.42676700	0.09051500	0.09981500
O	1.21305800	-1.06885500	0.95374400
O	2.48605400	-0.16730500	-0.74856000
O	0.84270300	-2.20085900	0.18732300
H	1.70799900	-2.61665400	0.02040100
C	0.20522300	0.43970700	-0.72247300
H	0.50841100	1.20737900	-1.43941900
H	-0.04963400	-0.44576500	-1.31091100
C	-0.99973300	0.93482000	0.10273400
H	-0.86608100	0.63952900	1.15270900
C	-2.29415700	0.28082800	-0.40469700
C	-1.08732800	2.48066000	0.10975500
H	-3.16383800	0.75069800	0.06309000
H	-2.37990400	0.44950100	-1.48395500
C	-2.34412800	-1.22104700	-0.10166900
O	-1.34288600	2.86803600	-1.24587200
C	-2.22029400	2.98486900	1.00595100
C	0.22356400	3.11579000	0.57739400
H	-1.54799500	-1.74819800	-0.64356200
H	-2.19913300	-1.41027000	0.96684900
C	-3.64226600	-1.87344300	-0.51376300
H	-1.35406100	3.83538700	-1.27014600
H	-3.20017900	2.70141800	0.61778400
H	-2.11741100	2.59545900	2.02332300
H	-2.18486100	4.07793000	1.05572000
H	0.46621100	2.80677200	1.59897200
H	1.05800700	2.85703800	-0.07968300
H	0.11948100	4.20578400	0.57021300
O	-4.24154400	-2.62128900	0.24462100
C	-4.16342600	-1.57812900	-1.89416700

H	-4.56713200	-0.55992000	-1.91262400
H	-3.35496300	-1.62004900	-2.62920000
H	-4.95497700	-2.28147400	-2.15308400
H	1.67546000	0.84357000	0.86047100
C	3.73885000	-0.37294400	-0.08518900
H	3.70824600	-1.31727300	0.47318100
H	3.90587000	0.44307200	0.63115200
C	4.83220100	-0.40777200	-1.13289500
H	4.60777300	-1.20500600	-1.84946900
H	4.82397500	0.53811400	-1.68442800
C	6.19696800	-0.63921700	-0.49317300
H	6.21942400	-1.58881000	0.05017100
H	6.98446000	-0.66594200	-1.24968700
H	6.43788400	0.15886700	0.21568200
O	-0.07671400	-1.07517600	3.05701000
H	-1.03183500	-0.93500900	2.92433500
H	0.04273100	-1.94396600	3.48262500
H	0.42725000	-1.05641900	2.12761200

Product of Reaction 1B

C	1.57565300	0.38929600	-0.10369400
O	0.91534900	-1.31139400	1.18854700
O	2.48938800	-0.26448300	-0.68054600
O	0.96114600	-2.40683000	0.28425900
H	1.62463800	-2.98025300	0.70108000
C	0.34828700	0.68647100	-0.86524400
H	0.60515300	1.53023800	-1.52350800
H	0.16573000	-0.16556900	-1.52483200
C	-0.85232000	1.04832800	0.03031000
H	-0.65892900	0.65038800	1.03257100
C	-2.16067500	0.40307000	-0.46166100
C	-0.99969400	2.57850900	0.18873100
H	-2.88260700	0.46505700	0.35887200
H	-2.57186900	0.97860000	-1.29655800
C	-2.01153000	-1.07788700	-0.87995900
O	-1.20686700	3.09379000	-1.13072300

C	-2.19752900	2.92785000	1.07171200
C	0.26478000	3.20254500	0.77986800
H	-1.77640000	-1.15710500	-1.94416500
H	-1.21346300	-1.55984100	-0.30981800
C	-3.29545000	-1.80487900	-0.57673900
H	-1.26911300	4.05653100	-1.05666100
H	-3.13696200	2.64206200	0.59396000
H	-2.12702800	2.42492900	2.04150200
H	-2.22041000	4.00797400	1.24698600
H	0.48340500	2.78594000	1.76801900
H	1.13217500	3.05342300	0.13034400
H	0.11875700	4.28171800	0.89104000
O	-3.48386700	-2.29222600	0.53358200
C	-4.34104700	-1.86126200	-1.64603700
H	-4.51329900	-0.85276900	-2.03619300
H	-3.96275700	-2.46505800	-2.47746300
H	-5.26795300	-2.28775400	-1.26373400
H	1.78950100	0.85305400	0.85973700
C	3.72021100	-0.58695500	0.04463500
H	3.63472300	-1.65318600	0.26687100
H	3.73189500	-0.00964600	0.97254100
C	4.89770800	-0.27676500	-0.84950800
H	4.78448400	-0.82487800	-1.78952000
H	4.89637100	0.79238200	-1.08179200
C	6.19672900	-0.67251900	-0.15342400
H	6.21001100	-1.74283000	0.07148300
H	7.05494800	-0.44932700	-0.79031700
H	6.32103000	-0.12656400	0.78620000
O	-1.42257300	-1.56961800	2.30534000
H	-1.71449800	-0.69267500	2.59104200
H	-2.12794300	-1.87875100	1.69880900
H	-0.00092500	-1.40481000	1.60590100

Reactant of Reaction 1C

C	1.45560100	-0.45821000	0.78262900
O	1.16852200	-1.88638900	0.49535100

C	0.07460900	0.13110500	1.06195800
H	0.23635300	1.07148700	1.60059400
H	-0.43426400	-0.54346200	1.75619700
C	-3.16060100	-0.54100900	0.02081200
C	-2.65105400	-1.70351400	0.87130600
H	-2.58285100	-1.40480300	1.92178900
H	-3.34915500	-2.54389700	0.79565800
H	-1.66569900	-2.05006300	0.54440600
C	-2.77740900	1.87482300	-0.66003600
H	-2.56456500	1.75433400	-1.73159400
H	-3.86746800	1.97731800	-0.58378800
C	-2.19080700	3.19102500	-0.25215000
H	-2.53363200	4.06726100	-0.83268700
O	-1.39193200	3.33875600	0.65427300
C	-2.25353000	0.70057200	0.16354000
H	-2.31728300	0.99356000	1.22070900
C	-0.77679400	0.41417800	-0.17720500
H	-0.34666600	1.27549900	-0.69527400
H	-0.72185300	-0.41499000	-0.88853300
O	2.37330300	-2.55460100	0.13872900
C	-3.33920600	-0.98758600	-1.43031800
H	-2.38881500	-1.27163700	-1.89175700
H	-3.99990200	-1.86057400	-1.46052400
H	-3.79559300	-0.19630200	-2.03074200
O	-4.43275800	-0.12569500	0.53853300
H	-5.04046600	-0.87145300	0.43455600
O	-0.00951500	-2.66113100	-1.52804800
H	-0.89021400	-3.03424500	-1.33681700
H	0.55215700	-3.37754200	-1.87765300
H	2.55281000	-3.08072000	0.93759600
H	0.43300600	-2.27001200	-0.63748800
C	3.37922600	0.18398800	-0.60250100
H	3.53061700	-0.17776100	-1.62506000
H	3.92107800	-0.48648000	0.06680800
C	3.87963200	1.61098800	-0.46576000
H	3.70365700	1.95517300	0.55953400

H	3.29038900	2.25444100	-1.12812900
C	5.36186200	1.70776100	-0.81060000
H	5.72139300	2.73522800	-0.71830000
H	5.96162900	1.08051800	-0.14368800
H	5.54739900	1.37723000	-1.83732900
C	2.33915200	-0.41099800	2.01536600
H	2.64677100	0.62156800	2.19318300
H	3.22610700	-1.03732300	1.92297900
H	1.75763500	-0.75618600	2.87266700
O	1.96404700	0.13094800	-0.36253400

Transition state (TS) of Reaction 1C

C	1.49620700	-0.13829000	0.86866400
O	1.12122700	-2.14788100	0.56370600
C	0.07098800	0.24110000	1.09212200
H	0.12403500	1.18841900	1.65065800
H	-0.36178000	-0.49083700	1.77899700
C	-3.14963300	-0.52449900	0.02831000
C	-2.63731400	-1.66959900	0.90061100
H	-2.56137500	-1.34671100	1.94360000
H	-3.33906800	-2.50878800	0.84949200
H	-1.65705700	-2.02988700	0.57436000
C	-2.76679000	1.88335600	-0.68631800
H	-2.55381700	1.74738800	-1.75587800
H	-3.85659500	1.98789200	-0.61074400
C	-2.17728800	3.20313700	-0.29454100
H	-2.52039300	4.07401100	-0.88261400
O	-1.37488100	3.35821900	0.60759100
C	-2.24619100	0.72043900	0.15495000
H	-2.31864800	1.02850600	1.20706100
C	-0.76439800	0.43412300	-0.16902700
H	-0.34241200	1.26792900	-0.73722500
H	-0.67559100	-0.44905600	-0.80967700
O	2.34932600	-2.64682600	0.05080000
C	-3.32442100	-0.98886300	-1.41718700
H	-2.36962900	-1.25330800	-1.88010400

H	-3.96555600	-1.87664700	-1.43834200
H	-3.80139700	-0.21174400	-2.02018800
O	-4.42251200	-0.10296800	0.53919500
H	-5.02886800	-0.85135000	0.44687100
O	-0.19583700	-2.70764800	-1.56549400
H	-1.06475800	-3.08121300	-1.35872500
H	0.31125500	-3.44424500	-1.93702000
H	2.58056700	-3.31506800	0.71676800
H	0.51573200	-2.31461400	-0.23843500
C	3.39867900	0.18155400	-0.58475200
H	3.45188800	-0.20763100	-1.60241600
H	3.91662200	-0.50205300	0.08669300
C	3.93216100	1.59639500	-0.49499700
H	3.80591200	1.96330900	0.52910000
H	3.34034300	2.24014900	-1.15285600
C	5.40388100	1.62914700	-0.89545400
H	5.79594900	2.64662500	-0.83693900
H	6.00469300	0.99638100	-0.23563700
H	5.53847500	1.27281400	-1.92094400
C	2.37433700	-0.37622900	2.04679800
H	2.91096800	0.55612400	2.25439700
H	3.10275000	-1.16430500	1.85595500
H	1.76082200	-0.63951900	2.90711900
O	1.97213000	0.19295400	-0.28059800

Product of Reaction 1C

C	1.52683000	0.09344300	0.96232300
O	0.99798900	-2.36365300	0.62890500
C	0.08590500	0.37284800	1.14210400
H	0.06027700	1.33042100	1.68824400
H	-0.29847100	-0.37386100	1.84468600
C	-3.12411300	-0.48718400	0.04121800
C	-2.61948300	-1.60796600	0.94843100
H	-2.53810000	-1.25322800	1.98069200
H	-3.32778300	-2.44287600	0.92510700
H	-1.64416800	-1.98679200	0.62944100

C	-2.72616600	1.89589400	-0.74113700
H	-2.50196500	1.72187900	-1.80287800
H	-3.81628100	2.00705500	-0.68155900
C	-2.13513600	3.22591400	-0.38965700
H	-2.47162400	4.07771500	-1.00863400
O	-1.33765600	3.40838300	0.51169300
C	-2.22138000	0.75970600	0.14537100
H	-2.31448400	1.10004000	1.18572600
C	-0.73223000	0.46405100	-0.13915500
H	-0.31030300	1.25626300	-0.76436200
H	-0.61532000	-0.46950100	-0.70138300
O	2.27909400	-2.64216300	0.06561800
C	-3.28264700	-0.98487900	-1.39473000
H	-2.31928000	-1.23288300	-1.84838300
H	-3.90098300	-1.88908100	-1.40222200
H	-3.77652000	-0.23087300	-2.01317300
O	-4.40155200	-0.05222000	0.52927600
H	-5.00756600	-0.80232600	0.44924300
O	-0.39870900	-2.88924000	-1.57020500
H	-1.26837900	-3.22819000	-1.31526800
H	0.06023200	-3.65156600	-1.95035900
H	2.48359800	-3.51135800	0.44530900
H	0.41021300	-2.53730300	-0.15554800
C	3.40680400	0.13255500	-0.54285100
H	3.40999300	-0.35311300	-1.51844900
H	3.90165800	-0.50701700	0.18663300
C	3.99220700	1.52819000	-0.59673900
H	3.89144600	1.99766400	0.38766200
H	3.41950900	2.12613600	-1.31157600
C	5.45961900	1.45732400	-1.00831000
H	5.89123800	2.45906600	-1.05666100
H	6.04068200	0.87163000	-0.29022500
H	5.56822200	0.99313200	-1.99267800
C	2.39510500	-0.20963800	2.12048400
H	3.18759200	0.54260500	2.19063900
H	2.86451100	-1.18626900	1.96567700

H	1.80437900	-0.21957800	3.03427600
O	1.98155800	0.23272500	-0.21329900

Reactant of Reaction 1D

C	1.44898500	0.75925400	0.70346700
O	2.68354300	0.14436200	0.89082000
O	1.56462500	1.60284300	-0.50318700
C	0.33217800	-0.22252600	0.35595900
H	0.46184800	-1.06799000	1.04215400
H	0.50307100	-0.59981500	-0.65712900
C	3.08716200	-0.81888600	-0.09219100
H	2.50169700	-1.73990000	0.02173800
H	2.90980000	-0.41943300	-1.09858900
C	4.56320700	-1.09323300	0.11603100
H	4.71616400	-1.44287600	1.14262300
H	5.11335500	-0.15201800	0.00767200
C	5.07661300	-2.12816100	-0.87900800
H	4.94077100	-1.78332000	-1.90865800
H	6.14075300	-2.32297700	-0.72740300
H	4.54179500	-3.07651400	-0.76937800
C	-3.54764400	0.04006600	-0.13821200
C	-3.55101900	1.34498100	-0.93678900
H	-3.06014800	1.20450500	-1.90570900
H	-4.58639600	1.65291600	-1.11597900
H	-3.05146200	2.15693500	-0.40245000
C	-2.15223500	-1.94565000	0.62353100
H	-1.92884100	-1.83482500	1.69327700
H	-3.14646100	-2.41214800	0.58109300
C	-1.22371200	-2.98993800	0.08017900
H	-1.14798600	-3.90785200	0.69223100
O	-0.60152800	-2.90585900	-0.96196800
C	-2.14287200	-0.60111900	-0.11117700
H	-1.88935900	-0.79561000	-1.16221100
C	-1.08370700	0.34243800	0.47095900
H	-1.31274700	0.53910400	1.52508300
H	-1.13587200	1.30281300	-0.04744700

O	2.59472700	2.56419900	-0.32306800
C	-4.12070700	0.27369200	1.25834300
H	-3.52260100	0.99636700	1.81974700
H	-5.13679000	0.67382700	1.17303800
H	-4.16902000	-0.65957200	1.82613300
O	-4.36471200	-0.91445700	-0.83141000
H	-5.26962200	-0.57200100	-0.82436500
O	-0.18570300	3.23204200	-1.13402500
H	-0.77633400	2.89883700	-1.83547200
H	0.28737600	4.01115300	-1.48089200
H	3.37258500	2.08422100	-0.65905800
C	1.18582200	1.62416000	1.91556100
H	0.34923000	2.29933400	1.72500400
H	0.94011000	0.98181100	2.76416000
H	2.07518000	2.20718600	2.15572300
H	0.51986600	2.48232000	-0.87214100

TS of Reaction 1D

C	1.43390600	0.52515800	0.97729800
O	2.66890500	0.18890000	0.89847200
O	1.44880800	1.73093600	-0.82049700
C	0.35669800	-0.37744300	0.48205900
H	0.42304400	-1.25823200	1.14144600
H	0.60533500	-0.72364500	-0.52404800
C	3.10493700	-0.88276600	0.01265300
H	2.63511000	-1.81325400	0.34238200
H	2.76342700	-0.63152700	-0.99599100
C	4.61269900	-0.93465700	0.09690500
H	4.90638400	-1.12570100	1.13353300
H	5.01074300	0.04486100	-0.18732700
C	5.15757100	-2.02246200	-0.82277900
H	4.87735900	-1.83360100	-1.86319500
H	6.24739800	-2.06153100	-0.76814600
H	4.76904600	-3.00535300	-0.54068700
C	-3.48743000	0.02923500	-0.15770800
C	-3.42564900	1.38803400	-0.85795400

H	-2.89557800	1.30663100	-1.81314400
H	-4.44494800	1.73458000	-1.05674300
H	-2.93146700	2.14470600	-0.24256900
C	-2.17320000	-2.03131600	0.54216900
H	-1.98411300	-1.98040000	1.62324900
H	-3.17832800	-2.46426000	0.44322500
C	-1.25605100	-3.07358000	-0.02408500
H	-1.25026500	-4.03478600	0.52228500
O	-0.57086700	-2.93954500	-1.02031000
C	-2.10216400	-0.65482600	-0.12578300
H	-1.82447700	-0.80586800	-1.17763800
C	-1.03994900	0.23837600	0.52400200
H	-1.31578400	0.43929300	1.56591100
H	-1.01085700	1.20001800	0.00453200
O	2.62169300	2.51431200	-0.64931400
C	-4.10942100	0.18205900	1.22927100
H	-3.51092300	0.83814900	1.86682200
H	-5.10533100	0.62765300	1.13249100
H	-4.21710900	-0.78703500	1.72376400
O	-4.30023700	-0.85175100	-0.94571600
H	-5.20165100	-0.50056700	-0.92702400
O	-0.46281700	3.47298700	-0.74195200
H	-1.13537500	3.17238800	-1.37035100
H	-0.16257000	4.32515100	-1.08847600
H	3.18987700	2.16289300	-1.35373100
C	1.17245600	1.62298900	1.93881600
H	0.32701600	2.23089800	1.61828100
H	0.91482600	1.15347400	2.89621200
H	2.06721300	2.23163500	2.06351700
H	0.72170600	2.43390700	-0.82322100

Product of Reaction 1D

C	1.44122200	0.47521300	1.04209200
O	2.67211600	0.19611000	0.89793800
O	1.43681000	1.82047300	-1.01752100
C	0.37619500	-0.39931500	0.49372400

H	0.43646700	-1.30984400	1.11405900
H	0.64618500	-0.70337700	-0.52106100
C	3.12369300	-0.88830300	0.02623700
H	2.64606400	-1.81220700	0.36163400
H	2.79174600	-0.63957400	-0.98571300
C	4.62922900	-0.93799600	0.13383500
H	4.90735000	-1.12667000	1.17498500
H	5.03238900	0.03932800	-0.14895500
C	5.18405700	-2.03033800	-0.77492400
H	4.91961100	-1.84344300	-1.81972300
H	6.27276100	-2.07105800	-0.70354900
H	4.78926500	-3.01141100	-0.49545200
C	-3.47307600	-0.01245600	-0.12483400
C	-3.43570500	1.37434400	-0.76857300
H	-2.91020900	1.34079900	-1.72926200
H	-4.46100600	1.71420300	-0.94665300
H	-2.94814200	2.11054500	-0.12394000
C	-2.12596100	-2.07572600	0.49185800
H	-1.92678400	-2.05983700	1.57235300
H	-3.12726400	-2.51605600	0.38809400
C	-1.20344300	-3.08832900	-0.11801300
H	-1.19576500	-4.07299700	0.38466300
O	-0.51468800	-2.90691300	-1.10429200
C	-2.07780900	-0.67691100	-0.12987900
H	-1.80996900	-0.78906900	-1.18904800
C	-1.02154300	0.21284400	0.53581100
H	-1.30351900	0.40376100	1.57785600
H	-0.98625900	1.17781800	0.02273500
O	2.53390100	2.64694500	-0.63416400
C	-4.08838100	0.07539800	1.27098000
H	-3.49418200	0.71170500	1.93214200
H	-5.08974000	0.51318400	1.19758400
H	-4.18298800	-0.91419700	1.72584500
O	-4.27844400	-0.87313100	-0.94233900
H	-5.18480500	-0.53671600	-0.90233100
O	-0.59697500	3.49291200	-0.67837600

H	-1.28007700	3.24378600	-1.31672700
H	-0.32407200	4.38179900	-0.94464300
H	2.94843500	2.84017200	-1.48988300
C	1.16054100	1.61100400	1.94269300
H	0.45396200	2.29254300	1.46255400
H	0.67249000	1.20933000	2.83884400
H	2.08108600	2.12588200	2.21103300
H	0.67108100	2.45386200	-0.94344800

Reactant of Reaction 2A

C	0.85260100	-0.84869100	0.47652100
H	0.13328500	-1.27843400	1.18009100
O	1.64116700	0.06623600	1.28834300
O	1.61821300	-1.91001400	0.02499500
O	2.12770000	-0.63550000	2.42300700
H	1.49520500	-0.35875200	3.10965600
C	0.16561000	0.00883300	-0.57141800
H	0.91634000	0.54761200	-1.15347100
H	-0.32054400	-0.68168500	-1.26896600
C	-0.87458700	0.97222800	0.04672300
H	-0.71454800	1.01364200	1.13318500
C	-2.30157700	0.46083400	-0.19381500
H	-3.02236500	1.14138000	0.26652400
H	-2.50971200	0.45745900	-1.27066500
C	-0.66857000	2.41754400	-0.46100700
O	-0.74128700	2.35042500	-1.89130800
H	-0.59568400	3.24533300	-2.22948500
C	-2.54794400	-0.92975600	0.37759100
H	-1.90086700	-1.69407200	-0.07221900
H	-2.32763000	-0.95585800	1.45435200
C	-3.97350400	-1.40255700	0.21123900
O	-4.84062400	-0.68136200	-0.25759500
C	-4.26986000	-2.81348600	0.64122300
H	-3.78103000	-3.50307600	-0.05493900
H	-3.85386300	-3.00536600	1.63378700
H	-5.34478300	-2.99458000	0.63744700

C	-1.74803700	3.36813700	0.05850600
H	-2.72414700	3.13008200	-0.36863300
H	-1.81954700	3.32313100	1.14962200
H	-1.49710500	4.39548600	-0.22439800
C	0.70400700	2.95176200	-0.04688100
H	0.80678400	2.96545300	1.04264100
H	1.50963800	2.34666100	-0.47049100
H	0.82586000	3.97537100	-0.41566900
O	3.56466900	1.61992600	1.17593900
H	4.39838700	1.11402100	1.14395200
H	3.58073500	2.26291100	0.44257900
H	2.75293000	0.96511800	1.06831600
C	2.38018300	-1.78478800	-1.19426200
H	1.70051300	-1.49515400	-2.00395200
C	3.50174600	-0.76542500	-1.06878500
H	3.12226400	0.25919800	-1.03165600
H	4.08775300	-0.96354600	-0.16469000
C	2.91460200	-3.17951500	-1.46184900
H	3.45909400	-3.19562100	-2.40871300
H	3.59718400	-3.48142300	-0.66158600
H	2.09624200	-3.90134600	-1.51631900
H	4.16290200	-0.84236100	-1.93606800

Product of Reaction 2A

C	0.58087900	-1.10222700	0.29061800
H	0.08286000	-1.28007800	1.24330100
O	1.75838200	0.35129200	1.62783400
O	1.46365900	-1.96630500	0.03252500
O	2.55231300	-0.62049900	2.30207000
H	2.46210100	-0.34502300	3.22857400
C	0.07794100	-0.08029900	-0.65036000
H	0.92236900	0.39251100	-1.15604700
H	-0.44664400	-0.64454100	-1.43905600
C	-0.86328800	0.94368600	0.01666500
H	-0.63700300	0.97749100	1.09035600
C	-2.33163500	0.53281600	-0.15224900

H	-2.97171300	1.24898600	0.36874400
H	-2.60052400	0.57734900	-1.21443700
C	-0.57116900	2.36089100	-0.52891300
O	-0.66560600	2.25722400	-1.95477000
H	-0.48700600	3.13523900	-2.32035800
C	-2.64378600	-0.85372100	0.39654600
H	-2.10059000	-1.65153400	-0.12800700
H	-2.34867300	-0.93720200	1.45191700
C	-4.11185400	-1.20758200	0.31706500
O	-4.93897000	-0.41848100	-0.11129800
C	-4.49913100	-2.58477800	0.78170000
H	-4.05362200	-3.32618700	0.11065900
H	-4.09921400	-2.77117700	1.78219100
H	-5.58354800	-2.69477600	0.78137000
C	-1.58489600	3.38631100	-0.02217400
H	-2.57850700	3.19514400	-0.43230600
H	-1.64425300	3.37375100	1.07041000
H	-1.27554100	4.38838600	-0.33573300
C	0.83673500	2.81681400	-0.14108800
H	0.94058400	2.87688200	0.94697100
H	1.60336000	2.14020200	-0.52870500
H	1.02691800	3.81043500	-0.55947200
O	3.85380500	1.79762300	0.97482600
H	4.52064800	1.10529300	0.86189800
H	3.80184900	2.22299700	0.10774000
H	2.46826100	0.96211500	1.27387300
C	2.27664900	-1.93634700	-1.21113600
H	1.63313700	-1.55208600	-2.00395600
C	3.45906600	-1.02972500	-0.94633300
H	3.14203200	-0.00653300	-0.73033800
H	4.04086400	-1.41035200	-0.10233500
C	2.64969800	-3.37710200	-1.46528400
H	3.24103800	-3.42670300	-2.38219700
H	3.24958700	-3.76625800	-0.63874400
H	1.75835800	-3.99496900	-1.59013600
H	4.09294700	-1.01861600	-1.83602200

Reactant of Reaction 2B

C	1.53331500	-0.13902400	0.02366500
O	1.22315200	-1.28371700	0.87341400
O	2.53370300	-0.51708100	-0.84988400
O	0.73181600	-2.36352200	0.09917600
H	1.55604200	-2.72602100	-0.27407100
C	0.33351600	0.34544900	-0.76062600
H	0.69635600	1.09383700	-1.47006900
H	-0.02370300	-0.49618100	-1.35960500
C	-0.79020200	0.94265400	0.10969900
H	-0.64904500	0.62190100	1.15191800
C	-2.16106500	0.42798800	-0.35555900
C	-0.72155200	2.48960900	0.13327100
H	-2.95862500	0.95175800	0.17926700
H	-2.28597500	0.65347400	-1.42052900
C	-2.33432000	-1.07644200	-0.11591400
O	-0.97907700	2.91733600	-1.20974100
C	-1.76963600	3.09456100	1.06915900
C	0.66201200	2.98383900	0.56232100
H	-1.64154600	-1.64915000	-0.74605900
H	-2.12175800	-1.33497300	0.92597000
C	-3.72053100	-1.57977400	-0.44116300
H	-0.90772600	3.88223200	-1.22106100
H	-2.78388500	2.91409400	0.70849800
H	-1.67564200	2.68545000	2.07962200
H	-1.62352200	4.17804200	1.12653900
H	0.90772700	2.63602600	1.57069800
H	1.44357200	2.65379600	-0.12767600
H	0.66766800	4.07873400	0.57183700
O	-4.32428600	-2.30885400	0.33184300
C	-4.32457700	-1.16027000	-1.75378000
H	-4.61765500	-0.10663300	-1.69031500
H	-3.59105300	-1.24511000	-2.56028700
H	-5.20528600	-1.76504000	-1.97031400
H	1.87364700	0.57838300	0.78127000

C	3.84738100	-0.63137100	-0.25484800
H	3.72514000	-0.97846000	0.77793400
C	4.59433500	-1.67660500	-1.05812000
H	4.07301400	-2.63702000	-1.01959900
H	4.67495000	-1.36156900	-2.10292500
O	-0.03399400	-1.21661300	2.99943400
H	-0.97870100	-1.00904800	2.88277700
H	0.03054000	-2.09550000	3.41612800
H	0.45590800	-1.22321000	2.06307900
H	5.60214000	-1.80925300	-0.65736500
C	4.52589300	0.72723300	-0.27738800
H	4.64679000	1.06619200	-1.31061400
H	3.93958700	1.47245800	0.26904800
H	5.51312300	0.66333200	0.18764200

Product of Reaction 2B

C	1.69741000	0.27840400	-0.10307400
O	0.98422200	-1.40009800	1.12112200
O	2.60737100	-0.35102700	-0.71695000
O	0.93290500	-2.46978200	0.18894000
H	1.53444200	-3.11570100	0.59359100
C	0.47024000	0.61609600	-0.85253900
H	0.76123300	1.42109500	-1.54342400
H	0.23875400	-0.24672400	-1.48298500
C	-0.69969800	1.06494700	0.04083400
H	-0.52612300	0.67350100	1.04992800
C	-2.05192100	0.49989600	-0.43380100
C	-0.74933200	2.60528200	0.17060400
H	-2.75914300	0.60912800	0.39447900
H	-2.43622100	1.09886200	-1.26484700
C	-2.00072700	-0.98740500	-0.85412200
O	-0.93123000	3.10611400	-1.15855700
C	-1.91759900	3.05021200	1.05024800
C	0.55529200	3.16203000	0.74179700
H	-1.80268500	-1.07870200	-1.92496300
H	-1.21381300	-1.51456700	-0.30988600

C	-3.31018500	-1.64681300	-0.50881800
H	-0.93200500	4.07202600	-1.10331300
H	-2.87618700	2.80526000	0.58809000
H	-1.86837900	2.57508200	2.03504200
H	-1.87610100	4.13477300	1.19163100
H	0.75512800	2.75309900	1.73708100
H	1.40747900	2.94599500	0.08994400
H	0.47713900	4.25030400	0.83083900
O	-3.48735300	-2.11899900	0.61002800
C	-4.38911600	-1.65924400	-1.54575900
H	-4.52609200	-0.64554200	-1.93639600
H	-4.06380400	-2.28343800	-2.38450700
H	-5.32283900	-2.04080200	-1.13372600
H	1.92966800	0.72132300	0.86681300
C	3.88769700	-0.60972500	-0.01196300
H	3.63465100	-0.67164900	1.04965300
C	4.37941200	-1.93637400	-0.53794500
H	3.64318600	-2.72185700	-0.35665700
H	4.58064300	-1.86910700	-1.61025300
O	-1.31716500	-1.54797800	2.29728800
H	-1.54901500	-0.67078900	2.63282800
H	-2.06581500	-1.79711800	1.71458100
H	0.06846200	-1.43682000	1.55829500
H	5.30682700	-2.19677500	-0.02315100
C	4.80422100	0.55654000	-0.30263100
H	4.98940700	0.63390200	-1.37706500
H	4.37104300	1.49319200	0.05835900
H	5.75600700	0.39313700	0.20806100

Reactant of Reaction 2C

C	1.53025400	0.11580900	0.95795400
O	1.50439300	-1.36305300	0.83303000
C	0.05258900	0.46708500	1.13106400
H	0.01020700	1.47101900	1.56659800
H	-0.35799300	-0.21707400	1.87907000
C	-2.89467100	-0.99843000	0.00680300

C	-2.15666000	-1.98688100	0.90926800
H	-2.17964400	-1.64400000	1.94813700
H	-2.64929600	-2.96352400	0.85754800
H	-1.11240700	-2.11794000	0.60725900
C	-3.03023700	1.41789200	-0.77116400
H	-2.76131200	1.30089800	-1.83036700
H	-4.11921100	1.28827800	-0.72586900
C	-2.75132900	2.84541700	-0.41398900
H	-3.25304600	3.60263100	-1.04420100
O	-2.03297300	3.19906700	0.50280500
C	-2.29546200	0.42004800	0.12068900
H	-2.46738300	0.73066600	1.16055200
C	-0.77790300	0.45138500	-0.15489300
H	-0.52511100	1.34113700	-0.73779500
H	-0.50315600	-0.40039400	-0.78490200
O	2.82433400	-1.89568500	0.80286300
C	-2.92464000	-1.51729800	-1.43046700
H	-1.92143900	-1.57517600	-1.86337000
H	-3.35360100	-2.52486700	-1.44457000
H	-3.54194200	-0.87672200	-2.06565700
O	-4.24217000	-0.86789400	0.48271400
H	-4.66897300	-1.72951500	0.37345900
O	0.89311800	-2.36161400	-1.33450500
H	0.02659200	-2.80939700	-1.31904100
H	1.58001700	-3.03488900	-1.49600700
H	2.90392500	-2.27132600	1.69730900
H	1.08304200	-1.88983400	-0.39451500
C	3.37199400	0.70721600	-0.57050600
H	3.95782600	0.27419900	0.24187500
C	3.75529400	2.16277400	-0.76614300
H	3.60371400	2.72938900	0.15649200
H	3.14339300	2.60942500	-1.55601700
C	2.33440500	0.47672900	2.19296300
H	2.39057500	1.56529500	2.25829100
H	3.34386900	0.06818600	2.18019700
H	1.81591800	0.09773500	3.07647200

O	1.96548100	0.67360800	-0.23030100
C	3.55847500	-0.12212500	-1.82862400
H	2.93596300	0.27612200	-2.63596900
H	4.60312300	-0.09141400	-2.14958900
H	3.27962300	-1.16412300	-1.65097800
H	4.80660500	2.24204100	-1.05499000

TS of Reaction 2C

C	1.54403500	0.33679200	0.95749500
O	1.51111800	-1.67527300	0.87609300
C	0.06931000	0.50474400	1.14708200
H	-0.04712100	1.48569500	1.63039200
H	-0.25560000	-0.23629300	1.88255400
C	-2.89804100	-0.95582600	0.02676800
C	-2.18834900	-1.95182100	0.94292600
H	-2.19763500	-1.59120400	1.97617100
H	-2.71190900	-2.91303000	0.90859100
H	-1.15080600	-2.12412100	0.64098600
C	-2.98922600	1.46071900	-0.75402500
H	-2.72509400	1.33347100	-1.81324800
H	-4.08012200	1.35204600	-0.70504100
C	-2.68048800	2.88343600	-0.40314200
H	-3.17208400	3.64840800	-1.03178200
O	-1.94828100	3.22534200	0.50718400
C	-2.27308300	0.45095500	0.13991900
H	-2.44120000	0.76535500	1.17914800
C	-0.75475500	0.45870900	-0.13616900
H	-0.49196300	1.32874800	-0.74456800
H	-0.46856200	-0.41943400	-0.72401000
O	2.84655300	-2.09593000	0.63338600
C	-2.92592800	-1.47659300	-1.40961000
H	-1.92408800	-1.52283600	-1.84628400
H	-3.34652700	-2.48771000	-1.42419800
H	-3.55080900	-0.84001800	-2.04154300
O	-4.24616600	-0.79641600	0.49182200
H	-4.68822800	-1.65052200	0.38448900

O	0.64998400	-2.44699700	-1.38633200
H	-0.19124400	-2.92064900	-1.30586300
H	1.29374900	-3.11946500	-1.65423000
H	3.05884200	-2.55462900	1.46335000
H	1.07788300	-1.95795900	-0.01229700
C	3.38775200	0.73565700	-0.58493400
H	3.96771700	0.20545700	0.16965600
C	3.73768700	2.20799100	-0.61908400
H	3.59848900	2.66825400	0.36204400
H	3.11257100	2.72700500	-1.35073100
C	2.42647600	0.41691700	2.15584700
H	2.73156200	1.46357400	2.26264200
H	3.31828800	-0.20090100	2.05817500
H	1.86313800	0.11586100	3.03838400
O	1.96409700	0.63550400	-0.22601200
C	3.50832700	0.04742600	-1.92654900
H	2.88971800	0.55659700	-2.67071900
H	4.54969100	0.08113600	-2.25495700
H	3.19581800	-0.99706500	-1.85475200
H	4.78477900	2.31976800	-0.91062900

Product of Reaction 2C

C	1.48680500	0.70115700	0.95658400
O	1.60122000	-1.88410500	1.06801400
C	0.01787800	0.67195600	1.13461900
H	-0.23099900	1.61500800	1.64508800
H	-0.18080600	-0.11348600	1.87426100
C	-2.81314900	-1.07834500	0.02021700
C	-2.01140700	-2.01524700	0.92238800
H	-2.01714500	-1.65034800	1.95432200
H	-2.46551400	-3.01157100	0.90614400
H	-0.97424600	-2.11637900	0.58887900
C	-3.10742600	1.32534100	-0.75034100
H	-2.84303800	1.21882300	-1.81172000
H	-4.18584500	1.13055100	-0.69203600
C	-2.90930300	2.76787700	-0.40154200

H	-3.46483800	3.49170400	-1.02560500
O	-2.19830000	3.16652200	0.50244200
C	-2.30673700	0.37488700	0.13698100
H	-2.49772100	0.66899800	1.17805000
C	-0.79428500	0.50825100	-0.14287500
H	-0.60635100	1.37633100	-0.78224100
H	-0.42349000	-0.36200000	-0.69274900
O	3.01357800	-2.00875000	0.90919000
C	-2.81091400	-1.59212800	-1.41926800
H	-1.81351700	-1.54703200	-1.86602800
H	-3.14091700	-2.63628500	-1.43552000
H	-3.49652000	-1.00977200	-2.04031300
O	-4.16460700	-1.03395200	0.49985900
H	-4.52887800	-1.92553200	0.40661400
O	0.83432200	-2.24549700	-1.46615900
H	0.05986200	-2.82352100	-1.40820700
H	1.51707700	-2.78955100	-1.88355700
H	3.17914800	-2.90766600	1.23530300
H	1.28950500	-2.02120800	0.13298000
C	3.33645100	0.77853700	-0.61864400
H	3.91859000	0.41243600	0.22725100
C	3.62101300	2.23489400	-0.91471700
H	3.44185600	2.85972200	-0.03642900
H	2.99330100	2.58193600	-1.73937100
C	2.38647700	0.82497800	2.12394300
H	2.98920800	1.73365300	2.02512200
H	3.06301800	-0.03507100	2.14914700
H	1.79926000	0.86423000	3.03953900
O	1.90347000	0.69437900	-0.23892300
C	3.47966400	-0.13344000	-1.81468700
H	2.81128100	0.18603900	-2.61860300
H	4.51041900	-0.07565200	-2.17218600
H	3.25986300	-1.16779100	-1.54453900
H	4.66958500	2.33235100	-1.20493500

Reactant of Reaction 2D

C	1.64497800	0.49670600	0.68454300
O	2.83308400	-0.22430000	0.74325300
O	1.74726600	1.44679500	-0.43942400
C	0.42612700	-0.35447300	0.33014900
H	0.49174300	-1.23900700	0.97346200
H	0.52810000	-0.69791500	-0.70499500
C	3.23280500	-1.00597000	-0.40669300
H	2.74183100	-0.59774000	-1.29704400
C	4.73664200	-0.82601100	-0.52818700
H	5.23383700	-1.22391400	0.36203200
H	4.99834600	0.23060700	-0.62724900
C	-3.40437000	0.35608800	-0.09425100
C	-3.25221900	1.68631500	-0.83544400
H	-2.77010600	1.53309400	-1.80710100
H	-4.24469600	2.11577300	-1.00678300
H	-2.67139700	2.41427300	-0.26359600
C	-2.25129600	-1.81557600	0.56587900
H	-2.02719600	-1.78318000	1.64036700
H	-3.29110400	-2.16513900	0.49401500
C	-1.44026300	-2.92731700	-0.02892100
H	-1.44127100	-3.86835900	0.55149200
O	-0.83676400	-2.86884400	-1.08425400
C	-2.08487400	-0.44627100	-0.10049200
H	-1.85190500	-0.61518300	-1.16060400
C	-0.92508400	0.33534100	0.52691200
H	-1.11316400	0.46525600	1.59937000
H	-0.89562000	1.33587100	0.09269200
O	2.84952800	2.32048900	-0.24068500
C	-3.94860500	0.59444900	1.31262900
H	-3.26608700	1.20936000	1.90543800
H	-4.90633300	1.12226200	1.24925300
H	-4.11454800	-0.35116200	1.83617000
O	-4.32623700	-0.46427800	-0.82620900
H	-5.18706600	-0.02374900	-0.79349700
O	0.08820500	3.19728600	-0.98093900
H	-0.54509400	2.89980700	-1.66134400

H	0.58882400	3.95264300	-1.34133300
H	3.50254800	1.94829900	-0.85878100
C	1.52044100	1.25427700	1.98881100
H	0.71869000	1.99248800	1.92442700
H	1.29137800	0.54810300	2.79007900
H	2.46043500	1.75768600	2.21615900
H	0.75878100	2.40808400	-0.74585600
H	5.11164200	-1.36305200	-1.40277800
C	2.86346100	-2.46992100	-0.21800400
H	3.26755900	-2.83211800	0.73239800
H	1.78373000	-2.63214200	-0.22661300
H	3.30102200	-3.06395800	-1.02530300

TS of Reaction 2D

C	1.62543200	0.25910300	0.87888700
O	2.82313400	-0.16919100	0.69771900
O	1.63367800	1.63986100	-0.71902500
C	0.43349800	-0.49203000	0.38618200
H	0.39769800	-1.36907600	1.05091400
H	0.61046400	-0.86827500	-0.62345600
C	3.19508600	-1.09037200	-0.39280500
H	2.56944200	-0.83065900	-1.25025200
C	4.64455100	-0.76381200	-0.67910800
H	5.26654200	-1.00163700	0.18815400
H	4.75825400	0.29459000	-0.92274900
C	-3.35832000	0.32696900	-0.15522200
C	-3.16751900	1.68291200	-0.83725700
H	-2.65144700	1.56654400	-1.79614400
H	-4.14952100	2.12920000	-1.02437300
H	-2.60118300	2.37771000	-0.21127300
C	-2.25380900	-1.85814300	0.51830700
H	-2.04705100	-1.82258400	1.59684900
H	-3.30014000	-2.18348700	0.43386400
C	-1.45948200	-2.99478700	-0.05171300
H	-1.54832500	-3.94768400	0.50185800
O	-0.77701600	-2.94071100	-1.05770300

C	-2.05138200	-0.49804800	-0.15630800
H	-1.81850300	-0.67837100	-1.21447700
C	-0.88333100	0.27836800	0.46291300
H	-1.11120100	0.50706500	1.51039100
H	-0.76863900	1.23042800	-0.06098900
O	2.86885400	2.31940000	-0.55189500
C	-3.94013600	0.52380500	1.24381100
H	-3.26086400	1.09299600	1.88401900
H	-4.87846600	1.08418000	1.17057000
H	-4.15387700	-0.43542900	1.72249800
O	-4.26958900	-0.45519500	-0.93977900
H	-5.13038700	-0.01512000	-0.90014800
O	-0.03418400	3.56295000	-0.39229400
H	-0.72871900	3.46090400	-1.05973700
H	0.41493000	4.38880000	-0.62421800
H	3.31943000	2.11161400	-1.38678300
C	1.50630900	1.23484100	1.99340800
H	0.70840400	1.95056200	1.79667700
H	1.24604900	0.66751600	2.89486400
H	2.45629200	1.74523800	2.14740400
H	0.96516200	2.39831900	-0.60840900
H	4.98395200	-1.36033100	-1.52858900
C	2.98418900	-2.52346000	0.05013800
H	3.51006400	-2.70406300	0.99143400
H	1.92857300	-2.77352100	0.17099800
H	3.40351400	-3.18442600	-0.71247800

Product of Reaction 2D

C	1.61895000	0.14212300	1.00040100
O	2.81323800	-0.18771600	0.73059300
O	1.67282900	1.71930600	-0.93277900
C	0.43753300	-0.55759900	0.44038800
H	0.37366900	-1.46496900	1.06456100
H	0.63534400	-0.89543900	-0.57851900
C	3.20082200	-1.09193200	-0.37997500
H	2.54804900	-0.84149600	-1.21854700

C	4.62887800	-0.70191900	-0.68472700
H	5.27696200	-0.92612100	0.16654400
H	4.69078600	0.36334300	-0.91735200
C	-3.33743400	0.32025100	-0.11786900
C	-3.13509700	1.71666300	-0.70723700
H	-2.60824300	1.66095400	-1.66602500
H	-4.11336000	2.17822200	-0.87598600
H	-2.57283800	2.36630500	-0.03122300
C	-2.24581100	-1.90248900	0.43815300
H	-2.05478300	-1.93110000	1.51987900
H	-3.29167800	-2.21869600	0.32054800
C	-1.44810000	-3.00790100	-0.18665700
H	-1.55608700	-3.99279500	0.30384000
O	-0.74438900	-2.89676000	-1.17306200
C	-2.03144600	-0.50568300	-0.15286500
H	-1.78678500	-0.62422000	-1.21704100
C	-0.86850400	0.23173400	0.52230300
H	-1.11459300	0.42248000	1.57318300
H	-0.71958600	1.20072600	0.03871300
O	2.81934100	2.42166000	-0.45510500
C	-3.94020600	0.42716900	1.28204300
H	-3.26813800	0.94801600	1.96928500
H	-4.87419400	0.99718100	1.23205300
H	-4.16664100	-0.56043000	1.69207800
O	-4.23694000	-0.40790600	-0.96523800
H	-5.09726300	0.03145400	-0.91191900
O	-0.15169500	3.57503800	-0.44465000
H	-0.85299300	3.49724300	-1.10692300
H	0.25735100	4.43448200	-0.61820400
H	3.29609300	2.61150600	-1.27863400
C	1.47736800	1.16792700	2.05451300
H	0.88139800	1.99425700	1.65406600
H	0.91443900	0.73428500	2.88808400
H	2.45200300	1.52017900	2.38612500
H	0.96422800	2.41020800	-0.82022700
H	4.97297500	-1.27285900	-1.54967400

C	3.04069400	-2.52988900	0.06243000
H	3.59090800	-2.70047300	0.99126200
H	1.99452300	-2.80972900	0.20049300
H	3.46350500	-3.17221400	-0.71392600

Reactant of Reaction 3A

C	0.96957800	-0.92474700	0.59583300
H	0.18693200	-1.45580400	1.14962700
O	1.70003500	-0.13740700	1.52918100
O	1.76278300	-1.93517300	0.03740800
O	2.07505800	-0.97558200	2.61734900
H	1.36672800	-0.79840400	3.25928200
C	0.38124900	0.07118600	-0.39147000
H	1.18526500	0.70236400	-0.77888600
H	-0.00105000	-0.49793100	-1.24574100
C	-0.74416800	0.92420400	0.23667600
H	-0.70344400	0.80996000	1.32931000
C	-2.12244900	0.43750100	-0.23119900
H	-2.90888100	1.02994500	0.24381200
H	-2.21503000	0.59507600	-1.31288300
C	-0.51903200	2.42917400	-0.03630500
O	-0.36780300	2.55618200	-1.45703000
H	-0.21587400	3.49273500	-1.64644900
C	-2.38610000	-1.02702000	0.09597600
H	-1.67054600	-1.70402900	-0.38891800
H	-2.28029100	-1.21361900	1.17405600
C	-3.76902700	-1.49064800	-0.29826800
O	-4.58867100	-0.72823300	-0.78689600
C	-4.08518900	-2.94344100	-0.06574600
H	-3.44909200	-3.55613200	-0.71254400
H	-3.85579800	-3.21801800	0.96763100
H	-5.13371700	-3.14431200	-0.28573100
C	-1.70154300	3.28397600	0.42158200
H	-2.58612100	3.09402600	-0.18975400
H	-1.94767400	3.08661500	1.46953600
H	-1.44533200	4.34420400	0.32581400

C	0.74828100	2.92657400	0.66169300
H	0.63840500	2.86133600	1.74839900
H	1.63200900	2.35428500	0.36921700
H	0.92061600	3.97582800	0.39985200
C	2.69518500	-1.57104300	-0.98419000
H	3.09375600	-2.52714300	-1.33753000
H	2.17194900	-1.10322200	-1.82735200
C	3.84355400	-0.68738700	-0.51583200
H	3.47043000	0.30854100	-0.25769400
H	4.26993200	-1.11870800	0.39692600
C	4.90539800	-0.57879500	-1.60613500
H	4.48074200	-0.16195600	-2.52510800
H	5.72702700	0.07005700	-1.29367800
H	5.32341400	-1.56118800	-1.84698800

H_2O Dimer for Reaction 3A-3D

O	-1.46242300	-0.10886700	0.02306000
O	1.35126100	0.02397200	-0.11683000
H	1.52633000	0.72033700	0.52981500
H	1.59577900	-0.78681800	0.34861700
H	-0.49029000	-0.06181200	-0.04454100
H	-1.74251900	0.80745500	-0.08373400

TS of Reaction 3A

C	1.24808700	-0.29681600	-0.23295500
O	2.47837300	-0.94319900	1.98890100
O	1.30375500	-2.34461700	-1.79398600
O	2.33056800	-3.04636100	0.66388600
H	2.33163100	-2.19188700	1.28750100
H	1.67548600	-2.70583400	-0.95461200
H	1.65333500	-3.62522600	1.03546700
H	2.19354000	-0.81589500	-0.14361800
O	1.25716700	0.83707400	-0.79036000
H	0.44704800	-2.77923200	-1.88712400
C	-0.01326200	-0.80218800	0.34403400
H	0.18372900	-1.82312100	0.66453300

H	-0.16309500	-0.21443100	1.25913800
C	2.54544000	1.39219500	-1.22033300
H	3.23605300	0.55732400	-1.36385100
O	3.74642500	-0.59222500	1.36562500
H	4.30158500	-1.36105200	1.56175200
H	2.33356700	1.87736400	-2.17279300
C	-1.25124300	-0.70225700	-0.57545100
H	-0.92869700	-0.94669600	-1.59473800
C	-2.27021200	-1.80224800	-0.18380200
C	-1.82492600	0.72437100	-0.63771900
H	-1.03839600	1.38632400	-1.00913700
H	-2.62102900	0.74859900	-1.38886300
C	-1.75594700	-3.19015000	-0.57805200
H	-0.77735000	-3.41117600	-0.14144300
H	-2.45812500	-3.95040400	-0.22072800
H	-1.69064600	-3.28276700	-1.66722500
C	-3.62861100	-1.57968300	-0.84793300
H	-4.28914500	-2.41966500	-0.60999500
H	-4.10363600	-0.66247400	-0.49260100
H	-3.52685000	-1.52420900	-1.93596600
O	-2.41318400	-1.74171500	1.24044700
H	-3.03873700	-2.43536400	1.49398800
C	-2.36441300	1.28301300	0.68531600
H	-3.15864600	0.65991400	1.09969700
H	-1.55595900	1.33357100	1.42764500
C	-2.89215000	2.69102300	0.54408700
C	-2.01296400	3.70406300	-0.14018800
H	-0.96631800	3.57429900	0.14833000
H	-2.07526500	3.55096200	-1.22333500
H	-2.35521200	4.71233700	0.09395800
C	3.02156000	2.36144900	-0.16089400
H	2.28761900	3.16613100	-0.05550100
H	3.07905400	1.82967300	0.79458000
C	4.38834700	2.92039800	-0.54322600
H	4.34898900	3.43294200	-1.50871800
H	4.73226400	3.63594800	0.20631500

H	5.12916200	2.11847200	-0.61251400
O	-3.99362800	2.99936200	0.97455800

Product of Reaction 3A

C	-1.39727300	-0.55363500	1.01612300
O	-1.10593300	0.52505900	1.87933800
H	-1.26630800	-1.45396400	1.62418600
O	-2.76377000	-0.57475100	0.65756400
C	-3.18824900	0.46010300	-0.22552000
H	-2.80672900	0.27826800	-1.23944100
H	-2.80293100	1.43266700	0.11133900
H	-0.94448000	1.31708700	1.34673300
C	-4.70506400	0.48081800	-0.23606500
H	-5.06162000	0.66220100	0.78346700
H	-5.06701400	-0.50942500	-0.53382500
C	-5.23998600	1.54730100	-1.18526300
H	-4.90124400	1.36582100	-2.20996700
H	-6.33248800	1.55811000	-1.18993400
H	-4.89529100	2.54326700	-0.89018400
C	-0.46031900	-0.57967900	-0.18459300
H	-0.82866200	-1.31899600	-0.90151200
H	-0.52037500	0.39196800	-0.68931700
C	0.99873400	-0.89066100	0.21875100
H	1.06951200	-0.85527400	1.31545000
C	1.96934900	0.16212800	-0.33395300
C	1.39353700	-2.33158300	-0.18137300
H	2.99942000	-0.11752800	-0.09714100
H	1.89567500	0.19531300	-1.42718500
C	1.72796800	1.55412700	0.23812900
O	1.27939500	-2.39645000	-1.60969200
C	2.82894100	-2.67077200	0.22446200
C	0.44821100	-3.35490200	0.45153900
H	0.75805100	1.96924200	-0.06810200
H	1.70296600	1.53491000	1.33611700
C	2.76737200	2.56665700	-0.18118100
H	1.51649300	-3.29612800	-1.87509200

H	3.55379200	-2.09337000	-0.35261600
H	2.99204200	-2.47809700	1.28928200
H	3.02075800	-3.73249400	0.03773300
H	0.51286600	-3.31912200	1.54348600
H	-0.59065500	-3.18724300	0.15588000
H	0.73186700	-4.36204400	0.12821700
O	3.64296600	2.29399300	-0.98807000
C	2.67428000	3.92967100	0.44971300
H	1.65416900	4.31520500	0.37149200
H	2.90739300	3.84280300	1.51598300
H	3.37652500	4.61658200	-0.02277200

H_2O_2-H_2O complex for 3A-3D

O	-0.79872400	0.76943400	0.00253100
H	0.17709000	0.61845900	-0.02474900
O	-1.26998300	-0.57536600	-0.09667000
H	-1.41772200	-0.81027000	0.83243000
O	1.77134900	-0.04546900	-0.08241800
H	1.53377700	-0.97179000	-0.22661500
H	2.08571700	-0.02519200	0.83139700

Reactant of Reaction 3B

C	-1.40525400	0.04986800	-0.30505400
O	-1.15536700	-0.98454900	-1.25623100
O	-2.48778200	-0.27531000	0.51289600
O	-0.86056200	-2.19427000	-0.56434400
H	-1.74665300	-2.56062300	-0.40171100
C	-0.20344700	0.34671700	0.57104900
H	-0.50962800	1.07126000	1.33022900
H	0.04590800	-0.57429800	1.10504100
C	1.00577700	0.88179100	-0.22442600
H	0.83544300	0.68768500	-1.29287700
C	2.29319300	0.14673800	0.17729000
C	1.14054600	2.41720000	-0.08605200
H	3.15639300	0.61335600	-0.30639700
H	2.44036400	0.24204900	1.25916100

C	2.26906200	-1.33370000	-0.22232300
O	1.39616500	2.67107000	1.30168700
C	2.29595600	2.97022600	-0.92270300
C	-0.14834300	3.12990700	-0.50043400
H	1.50534100	-1.87319600	0.35218200
H	2.02614800	-1.44813500	-1.28344200
C	3.57699500	-2.04123000	0.03739300
H	1.44894700	3.63096300	1.41204600
H	3.26314900	2.63752400	-0.54142100
H	2.20451800	2.66304900	-1.96897200
H	2.28136100	4.06442300	-0.88723600
H	-0.38309600	2.93244600	-1.55100800
H	-0.99787700	2.82352800	0.11538500
H	-0.01968400	4.21069100	-0.37952700
O	4.09593200	-2.74688200	-0.81512700
C	4.21425200	-1.85371800	1.38813600
H	4.64393200	-0.84755600	1.44313400
H	3.46632000	-1.93108000	2.18200700
H	5.00576300	-2.58915400	1.53325000
H	-1.65061700	0.88875500	-0.97315600
C	-3.72824600	-0.39112100	-0.18644200
H	-3.68582100	-1.23574900	-0.88689300
H	-3.90290400	0.52375600	-0.77063900
C	-4.83285800	-0.60041100	0.82950300
H	-4.60651000	-1.49813600	1.41486300
H	-4.83964300	0.24669100	1.52359200
C	-6.18983500	-0.74051600	0.14828600
H	-6.19795300	-1.59328300	-0.53750300
H	-6.98399800	-0.89247200	0.88288300
H	-6.43306700	0.15653300	-0.42939800

TS of Reaction 3B

C	0.53776900	-1.21493600	0.71494100
O	0.13904800	-1.63290100	-1.89710900
O	0.15703600	-3.40108600	1.90899400
O	-0.45288200	-3.74266000	-0.72479000

H	-0.18714800	-2.86716800	-1.26087500
H	-0.13045300	-3.63530400	0.99283400
H	0.15846300	-4.42209600	-1.03515600
O	1.39671800	-0.47622200	1.27537300
H	-0.66764600	-3.25259000	2.38914000
O	-1.07614200	-1.46138200	-2.67687300
H	-0.99691900	-0.54426400	-2.97468400
H	0.89499500	-2.04687200	0.12196800
C	-0.89995100	-0.90048100	0.87144100
H	-1.44046600	-1.78176600	0.52831800
H	-1.08967800	-0.74478700	1.94015200
C	-1.37281700	0.34818800	0.08449100
H	-1.20563900	0.12862600	-0.97193300
C	-0.58527800	1.61716400	0.46861900
H	-0.04511700	1.46232200	1.40799800
C	-2.89740300	0.53810700	0.23143600
O	-3.12221100	0.99986600	1.56731900
H	-4.07729200	1.11051000	1.67695500
C	2.81433700	-0.83774200	1.10043700
H	3.31687100	-0.32855500	1.92151800
H	2.88064300	-1.92038100	1.23402500
C	3.30865700	-0.38418900	-0.25531400
H	2.68601900	-0.83912500	-1.03575100
H	3.19911400	0.70159400	-0.33361700
C	4.76859700	-0.79206100	-0.43294500
H	5.14028100	-0.46251400	-1.40522200
H	5.39751800	-0.34152300	0.34026900
H	4.88350000	-1.87817400	-0.37483400
H	-1.27318600	2.44534600	0.65413600
C	-3.66862900	-0.76172900	-0.00220500
H	-4.73929500	-0.54612300	-0.07481100
H	-3.34791800	-1.23945200	-0.93351600
H	-3.52203600	-1.46250600	0.82298800
C	-3.37757100	1.58293800	-0.77885100
H	-3.24701400	1.20828700	-1.79894900
H	-4.44254300	1.78131800	-0.62099900

H	-2.83741000	2.52837800	-0.68323300
C	0.39845100	2.00591700	-0.64187400
H	1.01091200	1.15130900	-0.94701100
H	-0.18039900	2.32273000	-1.52135500
C	1.30402000	3.15455500	-0.27319300
C	0.66137100	4.37453900	0.32920000
H	-0.25716600	4.63182300	-0.20539400
H	0.38416500	4.15283500	1.36578300
H	1.36036700	5.21098400	0.31790600
O	2.51158000	3.10132300	-0.45713100

Product of Reaction 3B

C	-1.01088400	-0.99174000	-0.87367100
H	-0.17016500	-1.50008800	-1.36290200
O	-1.70474500	-0.21607100	-1.82815400
O	-1.80523400	-2.05503600	-0.39214100
C	-0.50670100	-0.04458400	0.20249500
H	-1.35446900	0.53165300	0.58264700
H	-0.13708000	-0.64279000	1.04271300
C	0.60626400	0.89375900	-0.31765300
H	0.62621700	0.83928000	-1.41533000
C	1.98177900	0.44749200	0.19744500
H	2.76225500	1.09995900	-0.20397200
H	2.01131800	0.55192300	1.28894100
C	0.29857900	2.37094900	0.02136500
O	0.07917000	2.41776000	1.43841800
H	-0.11387800	3.33794600	1.66616200
C	2.33342600	-0.98451000	-0.18598900
H	1.62619400	-1.71393700	0.23158300
H	2.29081300	-1.12739800	-1.27443800
C	3.70710100	-1.41107100	0.27533700
O	4.40391200	-0.69229200	0.97537600
C	4.18113100	-2.76488700	-0.17964000
H	3.40679300	-3.51765300	-0.01017700
H	4.36765500	-2.72792000	-1.25805000
H	5.09963300	-3.04047800	0.33877400

C	1.45827900	3.30216500	-0.33474900
H	2.32251800	3.12037800	0.30743600
H	1.76132000	3.17335100	-1.37849000
H	1.14735400	4.34294100	-0.19654800
C	-0.95791600	2.84930700	-0.70874200
H	-0.79742100	2.84598700	-1.79112300
H	-1.82609200	2.22403800	-0.48726300
H	-1.18953800	3.87490000	-0.40246200
C	-2.80040600	-1.76158600	0.58828900
H	-3.19699800	-2.74123500	0.87481500
H	-2.33835900	-1.32510500	1.48308800
C	-3.94517400	-0.88012000	0.10544100
H	-3.57943700	0.13039700	-0.10094000
H	-4.32623100	-1.28305000	-0.83967600
C	-5.05347300	-0.83122400	1.15290600
H	-4.67622300	-0.44037800	2.10349700
H	-5.87493400	-0.18730100	0.82973600
H	-5.46109700	-1.82951400	1.34087400
H	-1.91700600	-0.79448400	-2.57389000

Reactant of Reaction 3C

C	1.19900000	-0.54094300	0.59746700
H	0.52918700	-1.13261500	1.23246200
O	1.81865800	0.43821400	1.42402300
O	2.12461400	-1.46343800	0.09742100
O	2.36165900	-0.22466500	2.56123400
H	1.66216600	-0.09034200	3.22290300
C	0.42316200	0.25035500	-0.44483400
H	1.09792000	0.96255600	-0.92668100
H	0.11009700	-0.45160600	-1.22516400
C	-0.80518800	0.96745000	0.16029800
H	-0.70912100	0.96409100	1.25545400
C	-2.09980700	0.22313200	-0.19466300
H	-2.95637700	0.72251300	0.26563500
H	-2.25484800	0.26119600	-1.28004800

C	-0.84118500	2.45692400	-0.25164500
O	-0.76927300	2.47536200	-1.68421700
H	-0.77351800	3.40333100	-1.95819000
C	-2.10534400	-1.22773800	0.27014400
H	-1.30342000	-1.82161200	-0.18728800
H	-1.93529100	-1.29308100	1.35423900
C	-3.40427200	-1.94469800	-0.01467600
O	-4.36368300	-1.36866500	-0.50420000
C	-3.45418300	-3.41000200	0.32370600
H	-2.78882200	-3.95229500	-0.35612500
H	-3.08683500	-3.57846300	1.33955300
H	-4.47046200	-3.79019100	0.21943400
C	-2.12950300	3.14938700	0.19510000
H	-2.99412300	2.76494000	-0.34983100
H	-2.29910600	3.01236400	1.26758000
H	-2.05515100	4.22358200	-0.00352100
C	0.35290900	3.21404600	0.33301300
H	0.29707400	3.23381600	1.42560000
H	1.30697700	2.76627400	0.04428200
H	0.33964400	4.24800800	-0.02747600
C	2.96334400	-1.07948600	-1.00734900
H	2.32617700	-0.76066700	-1.84136200
C	3.93008300	0.03916400	-0.64244800
H	3.41618100	0.98677800	-0.46906400
H	4.48371800	-0.22830100	0.26338300
C	3.70027900	-2.34713600	-1.40156600
H	4.32255100	-2.16282100	-2.28050200
H	4.34524600	-2.67787800	-0.58148700
H	2.99358700	-3.14730100	-1.63504600
H	4.64497800	0.18053300	-1.45811000

TS of Reaction 3C

C	-1.53395100	-0.34507500	-0.30891100
O	-1.09612100	-1.35028700	-1.19945900
O	-2.55722800	-0.82647800	0.52788000
C	-0.40974700	0.15273700	0.58163800

H	-0.82459800	0.84757700	1.31612700
H	-0.04532100	-0.71161700	1.15049300
C	0.73163900	0.82042300	-0.21327400
H	0.55487400	0.65082800	-1.28462500
C	2.08752300	0.18655500	0.12991600
C	0.72222000	2.35468300	-0.02334300
H	2.89431000	0.73399000	-0.36668500
H	2.26080900	0.26532600	1.20945200
C	2.17013000	-1.27994300	-0.31259000
O	0.93247800	2.58809000	1.37580800
C	1.83187700	3.04256900	-0.82043600
C	-0.62499800	2.94839000	-0.44150800
H	1.46153700	-1.89329800	0.26082800
H	1.91729800	-1.38322100	-1.37211400
C	3.53236500	-1.89303200	-0.09388200
H	0.90795100	3.54573300	1.51209600
H	2.81901400	2.79545900	-0.42503200
H	1.79276800	2.75626300	-1.87586700
H	1.70818000	4.12872100	-0.75800700
H	-0.81031600	2.77660700	-1.50642600
H	-1.45382500	2.52351000	0.13115700
H	-0.61607400	4.02982000	-0.26907500
O	4.08680600	-2.53294000	-0.97531400
C	4.17941200	-1.69406700	1.25032800
H	4.53952000	-0.66198800	1.32259600
H	3.45374400	-1.84175000	2.05477000
H	5.02250900	-2.37581100	1.36345400
H	-1.91349000	0.45595500	-0.95710300
C	-3.81983300	-0.99929400	-0.13647700
H	-3.62574800	-1.30322800	-1.17273400
C	-4.55292900	-2.11359900	0.58618500
H	-3.97578700	-3.04096700	0.55018800
H	-4.71067700	-1.84054700	1.63440300
H	-0.68442200	-2.04604300	-0.66429400
H	-5.52798000	-2.28928100	0.12475900
C	-4.59541600	0.30926000	-0.11351300

H	-4.80130900	0.60092800	0.92113700
H	-4.03279900	1.11429700	-0.59578200
H	-5.54740800	0.19801200	-0.63983200

Product of Reaction 3C

C	-1.53395100	-0.34507500	-0.30891100
O	-1.09612100	-1.35028700	-1.19945900
O	-2.55722800	-0.82647800	0.52788000
C	-0.40974700	0.15273700	0.58163800
H	-0.82459800	0.84757700	1.31612700
H	-0.04532100	-0.71161700	1.15049300
C	0.73163900	0.82042300	-0.21327400
H	0.55487400	0.65082800	-1.28462500
C	2.08752300	0.18655500	0.12991600
C	0.72222000	2.35468300	-0.02334300
H	2.89431000	0.73399000	-0.36668500
H	2.26080900	0.26532600	1.20945200
C	2.17013000	-1.27994300	-0.31259000
O	0.93247800	2.58809000	1.37580800
C	1.83187700	3.04256900	-0.82043600
C	-0.62499800	2.94839000	-0.44150800
H	1.46153700	-1.89329800	0.26082800
H	1.91729800	-1.38322100	-1.37211400
C	3.53236500	-1.89303200	-0.09388200
H	0.90795100	3.54573300	1.51209600
H	2.81901400	2.79545900	-0.42503200
H	1.79276800	2.75626300	-1.87586700
H	1.70818000	4.12872100	-0.75800700
H	-0.81031600	2.77660700	-1.50642600
H	-1.45382500	2.52351000	0.13115700
H	-0.61607400	4.02982000	-0.26907500
O	4.08680600	-2.53294000	-0.97531400
C	4.17941200	-1.69406700	1.25032800
H	4.53952000	-0.66198800	1.32259600
H	3.45374400	-1.84175000	2.05477000
H	5.02250900	-2.37581100	1.36345400

H	-1.91349000	0.45595500	-0.95710300
C	-3.81983300	-0.99929400	-0.13647700
H	-3.62574800	-1.30322800	-1.17273400
C	-4.55292900	-2.11359900	0.58618500
H	-3.97578700	-3.04096700	0.55018800
H	-4.71067700	-1.84054700	1.63440300
H	-0.68442200	-2.04604300	-0.66429400
H	-5.52798000	-2.28928100	0.12475900
C	-4.59541600	0.30926000	-0.11351300
H	-4.80130900	0.60092800	0.92113700
H	-4.03279900	1.11429700	-0.59578200
H	-5.54740800	0.19801200	-0.63983200

Reactant of Reaction 3D

C	-1.48727100	-0.22980000	-0.25496100
O	-1.12783300	-1.27710000	-1.15925600
O	-2.53174200	-0.65792300	0.56635800
O	-0.76911100	-2.43176500	-0.40511600
H	-1.61234200	-2.66109400	0.02679000
C	-0.32609700	0.22409900	0.60677200
H	-0.71348600	0.91351300	1.36105700
H	0.04546900	-0.65013500	1.14860400
C	0.79263800	0.89910600	-0.21399400
H	0.61861900	0.69266700	-1.27970500
C	2.16539500	0.31407000	0.14706700
C	0.74413900	2.43822400	-0.06702400
H	2.95862300	0.88272500	-0.34722400
H	2.32540600	0.40914500	1.22714800
C	2.29509400	-1.15309600	-0.28035600
O	0.99023900	2.71446600	1.31809500
C	1.81019300	3.13137500	-0.91742200
C	-0.62962500	2.98957300	-0.45608600
H	1.59170400	-1.77956100	0.28353300
H	2.06171300	-1.27085600	-1.34323300
C	3.66950500	-1.72725600	-0.03696200
H	0.94246200	3.67438000	1.43093800

H	2.81614200	2.91576800	-0.55236700
H	1.74036600	2.82031900	-1.96418000
H	1.66391100	4.21562500	-0.87451400
H	-0.85307900	2.77475300	-1.50586400
H	-1.42704300	2.57397200	0.16624700
H	-0.63487200	4.07667800	-0.32450200
O	4.25332600	-2.36173200	-0.90344000
C	4.29163500	-1.49828600	1.31429100
H	4.62627100	-0.45717600	1.37976100
H	3.55794700	-1.65405400	2.10983000
H	5.14898000	-2.15827600	1.44758500
H	-1.81172200	0.55395500	-0.95304900
C	-3.81328400	-0.73027300	-0.09321200
H	-3.64311500	-1.00043800	-1.14253100
C	-4.59560800	-1.83255200	0.59293900
H	-4.07100700	-2.78836300	0.50616700
H	-4.72475200	-1.59796300	1.65405600
H	-5.58401600	-1.93446100	0.13811700
C	-4.50155800	0.62153900	-0.00488000
H	-4.67573600	0.88294300	1.04333300
H	-3.89224600	1.40627600	-0.46389400
H	-5.46416700	0.59174900	-0.52228600

TS of Reaction 3D

C	0.02455700	-1.43870700	-0.47085300
O	0.90948500	-1.37845900	2.05298300
O	1.27650000	-3.26100700	-1.67991700
O	2.30828700	-3.02990400	0.83155700
H	1.71690400	-2.34997900	1.39053500
H	1.75712000	-3.24445200	-0.81644300
H	2.15712200	-3.89024400	1.24224800
O	-1.15078700	-1.26233200	-0.90101100
H	1.85117500	-2.77398500	-2.28427500
O	1.97078800	-0.57080000	2.63217200
H	1.48873900	0.21482900	2.92626100
H	0.20356800	-2.28347000	0.18158300

C	1.08651300	-0.48128900	-0.85297200
H	2.03021200	-0.95537600	-0.58608800
H	1.03562400	-0.34803000	-1.94018600
C	0.97893300	0.90380400	-0.16594400
H	1.07913300	0.72484600	0.90649300
C	-0.37373500	1.59166000	-0.44020200
H	-0.88293900	1.11199900	-1.28211600
C	2.17826100	1.79588300	-0.55043300
O	1.97231600	2.18885200	-1.91113300
H	2.72548600	2.73889900	-2.16925200
C	-2.20195800	-2.23756000	-0.48411000
H	-1.75552900	-3.21941800	-0.66146000
C	-2.51381800	-2.01467200	0.97923100
H	-1.61466700	-2.08086200	1.59961100
H	-2.98266600	-1.03761800	1.12434200
H	-0.21397700	2.62955900	-0.74147100
C	3.51276200	1.05999500	-0.42187000
H	4.33706600	1.77351000	-0.51934300
H	3.59204900	0.57092100	0.55431400
H	3.62604700	0.30647300	-1.20474500
C	2.20617100	3.02689500	0.35882000
H	2.40967600	2.72759000	1.39178300
H	3.00444400	3.70233800	0.03482500
H	1.26296300	3.57879000	0.33549400
C	-1.26911300	1.55040700	0.80424200
H	-1.33554500	0.53668100	1.21230700
H	-0.81417300	2.18446000	1.57879400
C	-2.66202500	2.07766700	0.56519700
C	-2.78796900	3.39767100	-0.14611300
H	-2.05572600	4.11436200	0.23581700
H	-2.57338800	3.24791100	-1.21008100
H	-3.80036800	3.78710200	-0.03769500
O	-3.65119900	1.46159600	0.93514500
C	-3.36712700	-1.97063700	-1.40396800
H	-4.16877300	-2.67443100	-1.16992400
H	-3.73793800	-0.95328700	-1.24813400

H	-3.08116000	-2.09878700	-2.44964800
H	-3.21231000	-2.79061700	1.30194200

Product of Reaction 3D

C	1.27125700	-0.57406600	0.86326100
H	0.54272000	-1.18403900	1.41309900
O	1.84427200	0.36854900	1.74549600
O	2.22024400	-1.51832200	0.41731000
C	0.57785300	0.20090600	-0.24564800
H	1.29773300	0.89051000	-0.69406600
H	0.29401500	-0.50581700	-1.03330600
C	-0.66783000	0.96086600	0.26460900
H	-0.64570200	0.97144700	1.36355800
C	-1.95756900	0.24954700	-0.16829700
H	-2.82876700	0.77925900	0.22617900
H	-2.03756800	0.27725000	-1.26218300
C	-0.63610000	2.44481200	-0.16844800
O	-0.47160100	2.44076600	-1.59384600
H	-0.43920600	3.36449100	-1.87967100
C	-2.03824500	-1.19417600	0.31168800
H	-1.22209500	-1.81538600	-0.07921200
H	-1.94873700	-1.24939400	1.40592500
C	-3.33265700	-1.87950500	-0.05745900
O	-4.24055200	-1.28367500	-0.61670100
C	-3.44475400	-3.33988900	0.28750700
H	-2.74815900	-3.90564000	-0.33985200
H	-3.15399100	-3.50830500	1.32787200
H	-4.46098200	-3.69472400	0.11524600
C	-1.93029400	3.17974400	0.18456100
H	-2.76861300	2.81236700	-0.41090800
H	-2.17299100	3.06263700	1.24529100
H	-1.81227400	4.24868300	-0.02162700
C	0.53851300	3.17871200	0.48131600
H	0.41244700	3.21803100	1.56754800
H	1.49566600	2.69917700	0.26297000
H	0.57849500	4.20668600	0.10588800

C	3.12966200	-1.15047900	-0.63150800
H	2.55179000	-0.83899600	-1.51087300
C	4.07909600	-0.03097700	-0.22450600
H	3.55799900	0.91665200	-0.07620100
H	4.59047800	-0.29575400	0.70654000
H	2.17635100	-0.11749100	2.51312000
C	3.88688000	-2.42425300	-0.96596900
H	4.56707900	-2.25113100	-1.80342100
H	4.47485100	-2.75016400	-0.10211900
H	3.19416500	-3.22454300	-1.23818900
H	4.83150900	0.10821600	-1.00632500

Reactant of Reaction 4A

C	0.67311700	-0.83505700	0.75710400
H	0.11402200	-1.02064200	1.67258700
O	1.77063100	0.78213000	2.06419400
O	1.60909100	-1.66233300	0.57883100
C	0.20937100	0.16820900	-0.22917300
H	1.04766800	0.83427300	-0.44878700
H	0.00907400	-0.36698400	-1.16868900
C	-1.03126500	0.94093300	0.25226500
H	-1.00397500	0.99223500	1.34946600
C	-2.31624800	0.21575200	-0.17022300
H	-3.18178900	0.72993300	0.25449300
H	-2.41616300	0.26987300	-1.26113400
C	-0.97914300	2.40212200	-0.25157000
O	-0.74623200	2.32057700	-1.66370600
H	-0.71360300	3.22699800	-2.00146200
C	-2.37561100	-1.24055700	0.27354800
H	-1.58344800	-1.85596700	-0.17639000
H	-2.24338200	-1.33070600	1.36098800
C	-3.68721300	-1.91291700	-0.06764900
O	-4.60189500	-1.30095400	-0.59532400
C	-3.80250100	-3.37447000	0.26802900
H	-3.09726700	-3.93921600	-0.35018000
H	-3.52648400	-3.54425800	1.31237600

H	-4.81696900	-3.72735200	0.08269500
C	-2.29306100	3.13945500	0.00497800
H	-3.10411200	2.72095400	-0.59432700
H	-2.57209900	3.08948100	1.06192300
H	-2.17733600	4.19315400	-0.26787500
C	0.16058900	3.17657800	0.41222100
H	0.00467200	3.24347800	1.49286100
H	1.13644100	2.71865700	0.23108900
H	0.18872500	4.19338300	0.00726400
O	3.51221000	1.61446200	0.06714600
H	4.18783000	0.94956300	-0.12283000
H	3.05659900	1.73839300	-0.77659100
H	2.42002900	1.07903100	1.39012700
C	2.51959300	-1.61138000	-0.59341300
H	2.47447600	-0.59737400	-0.99286000
C	3.90130000	-1.93284100	-0.07011400
H	4.13534900	-1.23478500	0.74108300
H	3.90652500	-2.94425700	0.34576300
H	2.27548500	0.20614300	2.65463300
C	4.92305400	-1.80386300	-1.19638000
H	5.92391100	-2.04687300	-0.83438900
H	4.68656300	-2.48128400	-2.02200400
H	4.94276100	-0.78270700	-1.59078300
H	2.16415500	-2.33597800	-1.29592400

Product of Reaction 4A

C	0.71277700	-1.08706600	0.76143700
H	-0.13790000	-1.48203800	1.32266000
O	1.45862500	-0.30762300	1.72899400
O	1.43619700	-2.21159000	0.36241000
C	0.25106100	-0.15734300	-0.34343900
H	1.12126700	0.31931500	-0.80042900
H	-0.20259500	-0.77882600	-1.12297500
C	-0.76723000	0.89201900	0.15875700
H	-0.71102100	0.94045900	1.25492100
C	-2.20045800	0.49320600	-0.22135000

H	-2.90692600	1.22283000	0.18290300
H	-2.30541800	0.52039300	-1.31273400
C	-0.40210800	2.30758400	-0.34415300
O	-0.32086100	2.21303700	-1.77227800
H	-0.10573600	3.09573700	-2.10510700
C	-2.61105200	-0.88028500	0.29419700
H	-1.99382100	-1.68904100	-0.11838800
H	-2.49074500	-0.94482000	1.38484500
C	-4.04980600	-1.22786100	-0.01119200
O	-4.79601800	-0.44171600	-0.57389100
C	-4.51567800	-2.59652400	0.40585300
H	-3.97338700	-3.35085300	-0.17319800
H	-4.28257800	-2.76838000	1.46035500
H	-5.58685200	-2.70215300	0.23383200
C	-1.45999900	3.34440000	0.03430500
H	-2.39750400	3.16409200	-0.49531300
H	-1.65474300	3.33277600	1.11117600
H	-1.10566000	4.34401500	-0.23731400
C	0.95137100	2.75458900	0.21226100
H	0.93266700	2.78619700	1.30652400
H	1.75076500	2.08232900	-0.11128300
H	1.18785200	3.75775100	-0.15651200
O	3.40424300	1.11972100	1.80840700
H	4.22061400	0.66692500	1.53273700
H	3.33668800	1.94368900	1.29549900
H	2.52527400	0.46858600	1.63790400
C	2.31146200	-2.12256500	-0.76682800
H	2.61432400	-3.15783400	-0.94904300
H	1.75819600	-1.78177500	-1.64977800
C	3.54695200	-1.26425100	-0.54773500
H	3.26061800	-0.20905200	-0.47020300
H	4.01184300	-1.54781200	0.40403700
C	4.53059800	-1.44236800	-1.70033000
H	4.06666300	-1.17222000	-2.65415500
H	5.41289700	-0.81237500	-1.56628900
H	4.86440800	-2.48193200	-1.77233400

H	1.54598400	-0.82182300	2.54782400

Reactant of Reaction 4B

C	1.63550000	-0.21509200	-0.31819500
O	0.72104100	-1.88721200	1.03170600
O	2.39127400	-0.97311000	-0.98243200
C	0.40780200	0.28724900	-0.96023300
H	0.73444200	1.08644500	-1.64321300
H	0.02916000	-0.51325400	-1.60180500
C	-0.64638600	0.83476700	0.02202700
H	-0.46383100	0.38686400	1.00451500
C	-2.04820500	0.40880100	-0.43332300
C	-0.51162800	2.36460900	0.20398400
H	-2.79724300	0.90493000	0.19036600
H	-2.21517300	0.74222400	-1.46354200
C	-2.27149500	-1.10561100	-0.32061200
O	-0.77593900	2.93922000	-1.07920800
C	-1.51709100	2.89505500	1.22847800
C	0.89889100	2.75093800	0.65206600
H	-1.66219500	-1.64712900	-1.05589200
H	-1.99442700	-1.46552300	0.67416000
C	-3.70563100	-1.50580300	-0.57856000
H	-0.64458800	3.89481700	-1.00101200
H	-2.54267600	2.81044600	0.86528600
H	-1.43015200	2.35310400	2.17582500
H	-1.31484800	3.95297600	1.42283400
H	1.15131800	2.27824900	1.60616600
H	1.65296500	2.47912400	-0.09269500
H	0.95023900	3.83614100	0.78588600
O	-4.29954000	-2.24796800	0.18886600
C	-4.37596300	-0.94333000	-1.80210200
H	-4.59202700	0.11750800	-1.63356000
H	-3.71140000	-1.00508600	-2.66816100
H	-5.30943600	-1.47225700	-1.99420700
H	1.98033500	0.14622300	0.65065200
C	3.66630600	-1.37765400	-0.37354800

H	3.47485600	-1.55832200	0.68675100
O	-1.06040500	-1.18617600	3.03816800
H	-1.57556000	-0.42002200	2.75004600
H	-1.69587900	-1.91460700	3.00005000
H	0.08946200	-1.62721700	1.73825000
H	0.18040100	-2.36161000	0.38472600
C	4.70101500	-0.30108800	-0.61369600
H	4.78284800	-0.12002500	-1.68944200
H	4.36131200	0.62879600	-0.14308400
C	6.04563600	-0.73216200	-0.03634200
H	6.39728900	-1.65301600	-0.51018300
H	6.79765400	0.04208100	-0.20068200
H	5.97122200	-0.91078500	1.04010100
H	3.91198700	-2.31319800	-0.87460400

Product of Reaction 4B

C	1.44177100	-0.03706700	0.08693200
O	1.13780500	-1.23839600	0.84049100
O	2.51975100	-0.29093700	-0.74815100
C	0.25684800	0.38805600	-0.75413500
H	0.59040800	1.18220700	-1.42616700
H	-0.00229900	-0.46468700	-1.39307900
C	-0.95611700	0.85910500	0.07591500
H	-0.79403200	0.59511100	1.13035600
C	-2.23703300	0.14669100	-0.38151200
C	-1.08563300	2.40058400	0.04966400
H	-3.10731400	0.58200700	0.11786300
H	-2.37153600	0.30151800	-1.45809200
C	-2.20957000	-1.35279200	-0.06172500
O	-1.30734900	2.75792900	-1.32018000
C	-2.25882300	2.89341000	0.89866300
C	0.19703200	3.06804400	0.55006300
H	-1.42527800	-1.85536600	-0.64450000
H	-2.00012600	-1.52260300	0.99949000
C	-3.50134400	-2.05977900	-0.39834300
H	-1.34565700	3.72395800	-1.36202600

H	-3.21732500	2.59739100	0.46837000
H	-2.19488600	2.50563700	1.91992500
H	-2.23813800	3.98686400	0.94825100
H	0.39957400	2.78975000	1.58907200
H	1.06284200	2.80072000	-0.06140900
H	0.08042400	4.15596300	0.50755400
O	-4.03424900	-2.81455500	0.40130300
C	-4.10035500	-1.80744900	-1.75529200
H	-4.54446600	-0.80609000	-1.76770200
H	-3.32809700	-1.82970900	-2.52901400
H	-4.87540800	-2.54487300	-1.96440200
H	1.70031000	0.69091500	0.86607500
C	3.75605700	-0.48836100	-0.05594000
H	3.67760000	-1.36430800	0.59930300
H	3.96137000	0.39044900	0.57137800
C	4.85323700	-0.68672700	-1.08144600
H	4.59840100	-1.54826000	-1.70775000
H	4.89004400	0.19124000	-1.73493800
C	6.20285600	-0.90362800	-0.40548200
H	6.18045500	-1.78703700	0.23997500
H	6.99328400	-1.04797900	-1.14551900
H	6.47421700	-0.04282800	0.21330800
O	0.15237200	-0.97347100	3.02296900
H	-0.81729800	-1.03002200	2.97085200
H	0.45229200	-1.68699000	3.61267000
H	0.59376800	-1.11058600	1.99439600
H	0.70667500	-1.89746500	0.27014000

Reactant of Reaction 4C

C	1.69677800	0.60271600	-0.71170000
O	1.25936100	2.33311600	0.94921100
C	0.26058100	0.34009800	-0.96153500
H	0.23345700	-0.16786900	-1.93910300
H	-0.21730900	1.31099700	-1.11599900
C	-2.83233600	0.30049600	0.61716400
C	-2.41575500	1.75130000	0.38043400

H	-2.43840100	1.97990400	-0.69043600
H	-3.11642600	2.42074200	0.89025900
H	-1.41310400	1.95673700	0.76660800
C	-2.36957000	-2.12557000	0.01410000
H	-2.04098000	-2.52010700	0.98579100
H	-3.46069600	-2.24029600	0.00046900
C	-1.82379500	-3.04812000	-1.03179500
H	-2.10232700	-4.11234400	-0.92406900
O	-1.11999700	-2.69569600	-1.96035200
C	-1.94484300	-0.67277300	-0.18801400
H	-2.12713900	-0.42924700	-1.24361400
C	-0.43729700	-0.50567600	0.09700500
H	0.04487400	-1.48691200	0.13032400
H	-0.27593200	-0.04996500	1.08034800
C	-2.85003100	-0.00954400	2.11295800
H	-1.85199100	0.06667300	2.55326100
H	-3.49797700	0.70937900	2.62522100
H	-3.24085900	-1.01310400	2.29983700
O	-4.15134500	0.11471900	0.08409600
H	-4.74430800	0.69052200	0.58726000
O	-0.13016500	3.98831300	-0.78839000
H	-0.82002900	3.51603800	-1.27353400
H	0.52808800	4.20618500	-1.46211400
C	3.69662600	-0.04725800	0.42709700
H	3.81502000	0.92255400	0.91552100
C	2.44589500	1.57614700	-1.53371900
H	2.96581900	1.00260900	-2.31272600
H	3.19315000	2.10963700	-0.94605700
H	1.75601500	2.27441800	-2.00418100
O	2.27186100	-0.17840200	0.10692500
H	0.71461400	2.93615300	0.40038400
H	0.65907200	2.00761300	1.63272800
C	4.04792700	-1.20582800	1.32922700
H	5.08862000	-1.05839300	1.63401400
H	3.43572100	-1.14851800	2.23467200
C	3.87849600	-2.55635200	0.64181400

H	4.50126500	-2.61511300	-0.25595900
H	4.16801100	-3.36999400	1.31046700
H	2.83870300	-2.71949800	0.34431500
H	4.25128000	-0.07300900	-0.51381900

TS of Reaction 4C

C	1.59955000	0.34700300	-0.43648400
O	1.25479400	2.09733700	0.77875200
C	0.18219800	0.08396400	-0.81252500
H	0.23815400	-0.58276900	-1.68638100
H	-0.23859600	1.02596500	-1.17121100
C	-3.02280800	0.46955900	0.46095700
C	-2.46349800	1.84263100	0.09039200
H	-2.38180200	1.93088700	-0.99844400
H	-3.14204000	2.62357900	0.44924600
H	-1.48029200	2.01858000	0.53769700
C	-2.71367400	-2.04194100	0.21240600
H	-2.50540800	-2.32709800	1.25318100
H	-3.80527800	-2.07883500	0.10701900
C	-2.15822100	-3.13222700	-0.65152500
H	-2.53465500	-4.14956400	-0.43891700
O	-1.34865500	-2.95874700	-1.54384400
C	-2.15395500	-0.66257700	-0.12788100
H	-2.22522300	-0.54807100	-1.21817500
C	-0.66809200	-0.56307700	0.27630700
H	-0.27016900	-1.56116900	0.47839600
H	-0.56096900	0.00197700	1.20890200
C	-3.20064500	0.35803600	1.97431600
H	-2.24398300	0.41694400	2.50062400
H	-3.82781000	1.18315200	2.32789500
H	-3.69199400	-0.58091600	2.24199100
O	-4.30013100	0.31349400	-0.17306900
H	-4.88377900	0.99560200	0.18812200
O	0.04378900	3.74618900	-1.00305300
H	-0.59879300	3.24616600	-1.52489400
H	0.74019900	3.97342600	-1.63439300

C	3.44309100	-0.34204800	0.94234200
H	3.38985500	-0.71959500	1.96371600
H	3.74254200	0.70774700	0.96601300
C	4.34979600	-1.20200900	0.08623200
H	4.36027100	-0.82737700	-0.94160900
H	3.94828000	-2.21959400	0.06430300
C	5.76289600	-1.19634200	0.66254200
H	6.42572800	-1.81914600	0.05839600
H	6.17429200	-0.18292500	0.68164300
H	5.77044100	-1.58487600	1.68501100
C	2.46373600	1.10591500	-1.37693100
H	2.73758400	0.40878700	-2.17768200
H	3.37042500	1.49118700	-0.91387800
H	1.88570700	1.92163900	-1.81148400
O	2.06058700	-0.41816400	0.48319800
H	0.77134600	2.72182400	0.18493500
H	0.63065000	1.87227900	1.48320800

Product of Reaction 4C

C	-1.53554400	0.50064400	0.36431900
O	-1.42724100	1.68358500	-0.50265700
C	-0.14461600	0.07382700	0.81301100
H	-0.25976700	-0.68587600	1.59341800
H	0.32036600	0.94702400	1.28273400
C	3.08884500	0.57828800	-0.37601100
C	2.48681800	1.92715400	0.01808800
H	2.34574700	1.98127200	1.10265600
H	3.16811700	2.73055900	-0.28098000
H	1.52476800	2.10101400	-0.47511300
C	2.81033300	-1.94375200	-0.27958400
H	2.63526800	-2.16590800	-1.34158600
H	3.89940900	-1.97357400	-0.14594400
C	2.25004700	-3.09511500	0.49686000
H	2.64522100	-4.09133900	0.22540000
O	1.41862600	-2.99262300	1.38001200
C	2.22168900	-0.59535800	0.12847800

H	2.26692300	-0.54324200	1.22508400
C	0.74316700	-0.48556800	-0.30050200
H	0.36368400	-1.47178900	-0.58116100
H	0.67075900	0.12960600	-1.20608400
C	3.33057000	0.53161500	-1.88421100
H	2.39329300	0.57169500	-2.44623600
H	3.93680200	1.39330700	-2.18302600
H	3.86957100	-0.37654800	-2.16547100
O	4.34276000	0.42254600	0.30313400
H	4.93394000	1.11434600	-0.02546200
O	-0.48021100	3.60391600	0.60070800
H	0.23277200	3.32167800	1.19956600
H	-1.16263100	4.02452100	1.15166200
C	-3.40326900	-0.36768100	-0.90365100
H	-3.37794800	-0.69580100	-1.94775000
H	-3.68590200	0.69010300	-0.89863000
C	-4.39641400	-1.20843800	-0.12005800
H	-4.41178100	-0.88086000	0.92455500
H	-4.05455800	-2.24896600	-0.12776500
C	-5.79216800	-1.10343000	-0.72630500
H	-6.50900200	-1.71456000	-0.17305400
H	-6.14925400	-0.06890800	-0.71022000
H	-5.79371300	-1.44182800	-1.76713300
C	-2.40292400	0.94716600	1.52709300
H	-2.54573100	0.10614000	2.20787900
H	-3.37515100	1.30772300	1.18478300
H	-1.90090500	1.75550900	2.06541800
O	-2.06637900	-0.53003600	-0.40615000
H	-0.92241600	2.68475300	0.06998900
H	-0.91833000	1.45881900	-1.30138800

Reactant of Reaction 4D

C	-1.60266200	0.35072100	0.39954100
O	-1.44371000	-1.92303600	0.14543200
C	-0.30896100	0.68477200	-0.25788200
H	-0.35465400	1.76954800	-0.43380100

H	-0.31320100	0.21407500	-1.24561300
C	2.88036000	-0.70801700	-0.78922000
C	1.86964100	-1.57356900	-1.54100600
H	1.42584900	-1.00804700	-2.36644700
H	2.37895800	-2.44895900	-1.95733400
H	1.06826200	-1.92891300	-0.88668800
C	3.22412600	1.47223900	0.46916500
H	3.42951800	1.07961100	1.47488200
H	4.19735500	1.51895300	-0.03584900
C	2.76081400	2.88489000	0.64703800
H	3.45421200	3.55008600	1.19349400
O	1.70248500	3.32048200	0.23247200
C	2.22950600	0.60079400	-0.29413900
H	1.95187800	1.15132200	-1.20330900
C	0.95052900	0.35733600	0.53867400
H	0.96912800	0.98144400	1.43773900
H	0.90380600	-0.68170800	0.88117800
C	3.53558700	-1.49999300	0.34117400
H	2.82917100	-1.71727400	1.14703800
H	3.91261000	-2.45310300	-0.04465000
H	4.37843200	-0.94538700	0.76163300
O	3.89402900	-0.28733200	-1.71306800
H	4.36967300	-1.08121800	-1.99590500
O	0.54754200	-3.19012100	1.54143200
H	1.25182400	-3.39047600	0.90904100
H	0.95117400	-2.56039200	2.15440800
H	-0.71421500	-2.36522700	0.63460300
H	-1.22709800	-2.04102000	-0.78947700
C	-3.97425000	0.24012500	0.10191300
C	-1.74420200	0.22360700	1.86498400
H	-2.62635900	-0.34400700	2.15386200
H	-0.84883300	-0.22035900	2.29652900
H	-1.84149900	1.24969300	2.24735600
O	-2.61151800	0.46641200	-0.37412000
H	-4.02126700	-0.79065500	0.46303100
C	-4.89736900	0.47530900	-1.07050000

H	-4.75789300	1.49787500	-1.43397900
H	-4.62399800	-0.20729400	-1.88074200
H	-4.16485600	0.93672000	0.92160700
C	-6.34626700	0.25160400	-0.64862800
H	-6.63123900	0.93710600	0.15481500
H	-7.02120700	0.41847800	-1.49051700
H	-6.49725600	-0.77101400	-0.29076600

TS of Reaction 4D

C	-1.65937600	0.07734800	-0.01802800
O	-1.29739400	-2.09333700	-0.13708000
C	-0.32530200	0.52766000	-0.51084700
H	-0.45135500	1.59916100	-0.72320700
H	-0.14955300	0.04049600	-1.47440800
C	3.00693100	-0.58605300	-0.57634100
C	2.17860600	-1.55695800	-1.41756000
H	1.79383300	-1.05347400	-2.31007400
H	2.81232100	-2.39042100	-1.73836400
H	1.33596600	-1.97242200	-0.85673000
C	3.00718500	1.64587600	0.63365300
H	3.11261200	1.29795500	1.67072700
H	4.03035100	1.76026200	0.25314800
C	2.41396200	3.01874000	0.70088300
H	2.97112100	3.75094700	1.31357200
O	1.39163400	3.35633700	0.13304800
C	2.19270300	0.67435800	-0.21703500
H	1.99121600	1.17500800	-1.17374400
C	0.84185500	0.34113400	0.45442500
H	0.68802300	0.98972100	1.32275700
H	0.84472400	-0.68771600	0.82911300
C	3.57431500	-1.28764600	0.65740400
H	2.79139200	-1.53033500	1.38199900
H	4.06421500	-2.22109200	0.36076400
H	4.31666700	-0.65575300	1.15175500
O	4.09196500	-0.10337600	-1.38074000
H	4.64898500	-0.86520500	-1.59408200

O	0.56878500	-3.16298200	1.53547800
H	1.37380000	-3.29508600	1.01529900
H	0.82026200	-2.50092700	2.19414700
H	-0.60521900	-2.45875900	0.46241700
H	-0.95860700	-2.23381900	-1.03243300
C	-3.94589100	-0.28261500	-0.67553800
H	-4.29571700	-0.62164600	-1.65086400
H	-3.95419200	-1.12657700	0.01708800
C	-4.75245300	0.90230800	-0.18540200
H	-4.35197500	1.26018200	0.76810400
H	-4.65642100	1.71569000	-0.91083700
C	-6.21506500	0.49814700	-0.02315900
H	-6.31951300	-0.30751600	0.70941500
H	-6.81178300	1.34607700	0.31908600
H	-6.63304800	0.15004900	-0.97216800
C	-1.96675600	0.02407500	1.43041500
H	-2.90124800	-0.48397900	1.65909600
H	-1.14243300	-0.44601700	1.96573500
H	-2.03409100	1.06930500	1.76046700
O	-2.55931900	0.09306200	-0.93008200

Product of Reaction 4D

C	-1.55101000	-0.11022800	-0.00480400
O	-1.43397100	-1.56706400	-0.07106800
C	-0.25007200	0.52663700	-0.47759500
H	-0.44076600	1.59262600	-0.63659100
H	-0.02439200	0.09405500	-1.45918700
C	2.98655100	-0.78078600	-0.56244500
C	2.06577600	-1.68620200	-1.38069600
H	1.73498200	-1.17213800	-2.28826700
H	2.61290800	-2.58780800	-1.67554000
H	1.18287200	-1.99852700	-0.81316200
C	3.20572400	1.46036700	0.61412200
H	3.28291800	1.12719800	1.65859100
H	4.23367600	1.47026100	0.22941800
C	2.74531700	2.88501900	0.65268000

H	3.37372400	3.57446900	1.24602000
O	1.75607300	3.30551900	0.08162300
C	2.29567400	0.55483700	-0.21188300
H	2.12673400	1.05899800	-1.17313700
C	0.93067900	0.35658800	0.48019100
H	0.82303700	1.07580000	1.29850000
H	0.89717000	-0.63254300	0.94737700
C	3.50215000	-1.52481900	0.66933100
H	2.69626600	-1.75204600	1.37429400
H	3.95565800	-2.47252700	0.36084200
H	4.26260200	-0.93768800	1.19051800
O	4.10359700	-0.40953100	-1.38266000
H	4.58361000	-1.22181800	-1.59694700
O	-0.19659000	-2.84439900	1.56185000
H	0.73933900	-2.93188100	1.30675500
H	-0.21209300	-2.38853700	2.42210300
H	-0.75433300	-2.22138100	0.79730500
H	-1.16814700	-1.82218200	-0.97183100
C	-3.86073300	-0.18590300	-0.73699200
H	-4.21620700	-0.54501000	-1.70847900
H	-3.87917100	-1.03716400	-0.04844800
C	-4.75188500	0.94190500	-0.24468500
H	-4.38256100	1.30720800	0.71938900
H	-4.68260900	1.77465600	-0.95288100
C	-6.19810000	0.47558800	-0.11355400
H	-6.28139500	-0.34428800	0.60679200
H	-6.84506000	1.28783100	0.22588700
H	-6.58284400	0.11763100	-1.07368800
C	-1.91371400	0.20906800	1.43290600
H	-2.86473000	-0.24810800	1.71313500
H	-1.13982400	-0.16964000	2.10513500
H	-1.98326800	1.29272500	1.54979400
O	-2.51488900	0.26265100	-0.94638900

Reactant of Reaction 5A

C	-0.92360700	-0.88483100	-0.68424700

H	-0.12077800	-1.31575800	-1.28708800
O	-1.67582800	-0.06620000	-1.61300500
O	-1.67713600	-1.97771300	-0.26030500
C	-0.36966800	0.01558900	0.40323500
H	-1.19396400	0.55698700	0.87340600
H	0.05506600	-0.63255800	1.17753800
C	0.71330900	0.98550500	-0.12040700
H	0.59661400	1.09140100	-1.20799400
C	2.12324200	0.44171900	0.15420100
H	2.86617800	1.11267100	-0.28457800
H	2.30113500	0.43672000	1.23673100
C	0.51845800	2.40320400	0.46740600
O	0.46815600	2.23966100	1.89119000
H	0.35387500	3.11973200	2.27708300
C	2.37617800	-0.95234100	-0.40482400
H	1.72492000	-1.71450400	0.04252000
H	2.17510400	-0.98756700	-1.48502700
C	3.80027300	-1.42216000	-0.21427200
O	4.66038200	-0.69739000	0.26169100
C	4.10354000	-2.83591100	-0.63009400
H	3.61121400	-3.52062900	0.06844100
H	3.69583700	-3.03775000	-1.62408300
H	5.17885500	-3.01419500	-0.61617900
C	1.67062300	3.34215900	0.10805700
H	2.59710700	3.03679200	0.59824600
H	1.83523100	3.36719000	-0.97363200
H	1.43065700	4.35753500	0.43976900
C	-0.79142100	3.02455300	-0.01899400
H	-0.77477000	3.15245900	-1.10701900
H	-1.65635600	2.41491600	0.25468100
H	-0.92011000	4.01158500	0.43680800
O	-3.37898800	1.63649900	-1.58664000
H	-4.01995700	1.63127300	-0.85473200
H	-2.95929000	2.51470500	-1.59797400
H	-2.60192200	0.84971200	-1.46088800
C	-2.53132500	-1.86675000	0.89504000

H	-1.92676800	-1.53969200	1.74917800
C	-3.67938200	-0.89322100	0.67622200
H	-3.33007400	0.14205300	0.65982400
H	-4.19098100	-1.11644800	-0.26538600
H	-1.90491100	-0.60136200	-2.39010100
C	-3.03058300	-3.27769700	1.14725200
H	-3.63766400	-3.30291300	2.05512000
H	-3.64500000	-3.61646000	0.30740500
H	-2.19145600	-3.96694000	1.26839000
H	-4.39830100	-0.98672000	1.49464000

Product of Reaction 5A

C	0.79740800	-0.83104500	0.77273200
H	0.25642100	-0.93948000	1.71119500
O	2.07601100	0.72145700	1.99061000
O	1.65119900	-1.74235100	0.59168900
C	0.38863900	0.17938700	-0.23040300
H	1.27351300	0.76233700	-0.49832400
H	0.11009900	-0.36377700	-1.14507700
C	-0.76269300	1.07217000	0.26501200
H	-0.69297500	1.15342900	1.35852200
C	-2.11972000	0.45057500	-0.09228300
H	-2.92203800	1.05126200	0.34290500
H	-2.25236500	0.48085800	-1.18062400
C	-0.60131700	2.50750900	-0.28761900
O	-0.42553600	2.36390500	-1.70307100
H	-0.32601000	3.25351500	-2.07108100
C	-2.28996600	-0.98105100	0.40042000
H	-1.57037600	-1.67682500	-0.05431400
H	-2.12840100	-1.05015100	1.48543600
C	-3.66608400	-1.54529800	0.12346000
O	-4.54189300	-0.87120900	-0.39436700
C	-3.89621800	-2.98043700	0.51014300
H	-3.26461500	-3.62311800	-0.11155900
H	-3.59991200	-3.14275600	1.55010800
H	-4.94333300	-3.24808800	0.36895900

C	-1.83649100	3.36482000	-0.01336100
H	-2.70115500	3.00165400	-0.57244900
H	-2.08195500	3.37098300	1.05303600
H	-1.63918300	4.39569300	-0.32399600
C	0.62375600	3.19810400	0.31376600
H	0.51185100	3.31051200	1.39608300
H	1.54924600	2.65096600	0.11654500
H	0.72606800	4.19602200	-0.12483100
O	3.81294800	1.33774800	-0.08720400
H	4.42123100	0.61066500	-0.27690700
H	3.34081500	1.47615800	-0.91953000
H	2.72496000	0.93995500	1.28679200
C	2.52143800	-1.80649300	-0.61011600
H	2.55076700	-0.80475700	-1.04077400
C	3.87844600	-2.20363500	-0.07852900
H	4.22343700	-1.48590200	0.67016700
H	3.83771800	-3.19945000	0.36991400
H	2.54909600	0.12109100	2.58307000
C	1.89806600	-2.81109500	-1.55213700
H	2.52620800	-2.88945900	-2.44243500
H	1.84120200	-3.79194900	-1.07368600
H	0.89668100	-2.49784500	-1.85717000
H	4.58959200	-2.21900200	-0.90773500

Reactant of Reaction 5B

C	1.77096800	0.20228900	-0.17422300
O	1.04497000	-1.52871800	1.21537900
O	2.66319400	-0.46177800	-0.76578300
C	0.51715300	0.48752200	-0.89421900
H	0.75700400	1.29673500	-1.60098300
H	0.29395100	-0.38722200	-1.51130300
C	-0.65510100	0.91257300	0.01191200
H	-0.45908900	0.53930500	1.02259400
C	-1.95367800	0.26270900	-0.48332800
C	-0.75673200	2.45171100	0.12330400
H	-2.79858600	0.66735300	0.08110500

H	-2.11498100	0.52318600	-1.53524700
C	-1.95685700	-1.26186800	-0.30539600
O	-1.03677200	2.92509900	-1.19734000
C	-1.88050500	2.86898600	1.07458400
C	0.55665100	3.06341200	0.61365000
H	-1.23757500	-1.73700700	-0.98510300
H	-1.68108400	-1.53355600	0.71735000
C	-3.30126100	-1.88269100	-0.60638700
H	-1.05184000	3.89204700	-1.16105300
H	-2.86247100	2.61652700	0.67097700
H	-1.76328100	2.38701600	2.05064700
H	-1.84657400	3.95256800	1.22482100
H	0.82678500	2.67490100	1.60033600
H	1.37968000	2.87594600	-0.08252400
H	0.44061400	4.14879400	0.69544400
O	-3.81783600	-2.67194300	0.16995700
C	-3.98361700	-1.47948000	-1.88489600
H	-4.36198100	-0.45668300	-1.77909100
H	-3.27374500	-1.47800300	-2.71651800
H	-4.81788900	-2.14978000	-2.09160500
H	2.00873300	0.65208400	0.78976700
C	3.95343600	-0.73235600	-0.07931100
H	3.82337100	-0.40300000	0.95498700
C	4.15845200	-2.22628400	-0.16285300
H	3.34292600	-2.75728000	0.33199900
H	4.22266600	-2.54326400	-1.20678700
O	-0.92070300	-1.01713500	3.10358000
H	-1.52769100	-0.34963800	2.75515100
H	-1.43911100	-1.83337600	3.07364600
H	0.34663600	-1.33623500	1.87943700
H	0.61397900	-2.10578600	0.56949500
H	5.09674100	-2.47779400	0.33720200
C	5.00100400	0.08561000	-0.79719700
H	5.06320900	-0.21479900	-1.84606900
H	4.76866400	1.15117700	-0.73684700
H	5.96864000	-0.09122900	-0.32199900

Product of Reaction 5B

C	1.52810000	-0.30896700	-0.00103400
O	1.09947000	-1.48419500	0.73810100
O	2.54481300	-0.67992600	-0.86538700
C	0.38249600	0.27524900	-0.79944000
H	0.79399300	1.04038300	-1.46175800
H	0.00466100	-0.52138000	-1.45124700
C	-0.73934400	0.86924200	0.07846500
H	-0.57361000	0.57203500	1.12394700
C	-2.11032100	0.31904300	-0.33911100
C	-0.68479900	2.41495800	0.07566000
H	-2.90233100	0.82999500	0.21600200
H	-2.27534800	0.52610300	-1.40258900
C	-2.23516500	-1.18527000	-0.06782000
O	-0.90084200	2.81767200	-1.28213800
C	-1.76613900	3.03069600	0.96507100
C	0.68367700	2.91474200	0.54539200
H	-1.55138100	-1.75007400	-0.71677300
H	-1.97806100	-1.41836200	0.97081600
C	-3.61755900	-1.73064600	-0.33772800
H	-0.85251200	3.78377900	-1.30549700
H	-2.76436500	2.85811400	0.55830200
H	-1.72157900	2.62114000	1.97883000
H	-1.61321500	4.11291600	1.02859000
H	0.87799200	2.60260600	1.57643600
H	1.49350700	2.54808800	-0.09153900
H	0.70179300	4.00905100	0.51356100
O	-4.17557000	-2.46062100	0.46785800
C	-4.27513000	-1.35010500	-1.63629800
H	-4.59584900	-0.30412900	-1.57992300
H	-3.56601700	-1.42762300	-2.46506300
H	-5.14492200	-1.98245500	-1.81462000
H	1.88025900	0.36554700	0.78803700
C	3.82281900	-0.89578900	-0.22841800
H	3.64228700	-1.32731300	0.76360300

C	4.57262600	-1.89321300	-1.08871900
H	4.01907500	-2.83243900	-1.16212400
H	4.71376400	-1.48857500	-2.09565800
O	0.20510600	-1.18562100	2.95080500
H	-0.74310500	-0.97067900	2.92323800
H	0.29426900	-2.01604700	3.44960000
H	0.59753600	-1.32759300	1.89369400
H	0.59348900	-2.08458100	0.16451500
H	5.55544300	-2.09788800	-0.65726100
C	4.55527300	0.42947500	-0.10058500
H	4.73875800	0.85005000	-1.09391300
H	3.97623700	1.15183100	0.48271800
H	5.51621300	0.28252500	0.39955500

Reactant of Reaction 5C

C	1.74034100	0.18677800	-0.67188100
O	1.51933100	2.05960600	0.84394800
C	0.28689000	0.07477700	-0.95502500
H	0.21930500	-0.52545000	-1.87587600
H	-0.06508900	1.07482400	-1.22012900
C	-2.81351200	0.60557100	0.50151700
C	-2.19363400	1.95306000	0.13483800
H	-2.16141200	2.06719600	-0.95420300
H	-2.80534500	2.76205700	0.54759100
H	-1.18068600	2.05925300	0.53461800
C	-2.67449400	-1.91107400	0.17588900
H	-2.41814300	-2.24015700	1.19266300
H	-3.77080800	-1.87528900	0.14272700
C	-2.24903600	-3.00695600	-0.75215600
H	-2.67477200	-4.00492400	-0.54073500
O	-1.49198800	-2.85676200	-1.69347100
C	-2.04986900	-0.55964700	-0.16398300
H	-2.17535800	-0.41064600	-1.24509500
C	-0.53988600	-0.56620600	0.15430700
H	-0.19510200	-1.59450700	0.29372100
H	-0.33914000	-0.04395200	1.09653800

C	-2.91511700	0.46193900	2.01892400
H	-1.92934300	0.44723700	2.49192000
H	-3.46946800	1.31337900	2.42751100
H	-3.44863100	-0.45273100	2.29001500
O	-4.13126500	0.54467200	-0.06266900
H	-4.65521900	1.23762500	0.36362200
O	0.35594900	3.78814200	-0.98141200
H	-0.28382800	3.30080700	-1.51769000
H	1.06077200	4.01674100	-1.60240900
C	3.59646800	-0.62504000	0.66796300
H	3.94472200	0.40878500	0.65921500
C	4.38504200	-1.50315000	-0.27757500
H	4.39402500	-1.10302700	-1.29364900
H	3.96432000	-2.51183900	-0.29079100
C	2.59361900	1.01172300	-1.55603700
H	2.79615200	0.39763900	-2.44322100
H	3.54038400	1.30263000	-1.10600800
H	2.03311300	1.89182400	-1.87123700
O	2.18534100	-0.59996300	0.21789200
H	1.07319800	2.70393100	0.25413100
H	0.87364400	1.86686400	1.53635800
C	3.53050400	-1.15089600	2.08280500
H	3.13283100	-2.16896000	2.09430000
H	4.53948600	-1.16263600	2.50068500
H	2.90172700	-0.50872300	2.70347700
H	5.41580900	-1.55877400	0.08012500

TS of Reaction 5C

C	1.72914800	0.23521400	-0.62358600
O	1.56848000	1.92092900	0.71717700
C	0.28090900	0.07627200	-0.93937700
H	0.25355800	-0.55814300	-1.83830100
H	-0.09656900	1.05489700	-1.24348400
C	-2.82488200	0.62371300	0.49904400
C	-2.18655500	1.96760300	0.15006400
H	-2.14665600	2.09239700	-0.93750600

H	-2.79091200	2.77959100	0.56765600
H	-1.17406600	2.05719100	0.55607500
C	-2.70003400	-1.89303000	0.16839000
H	-2.45665600	-2.22380500	1.18780100
H	-3.79581000	-1.85127600	0.12344700
C	-2.27069600	-2.99155000	-0.75468500
H	-2.70375900	-3.98716800	-0.54692400
O	-1.50303500	-2.84619100	-1.68811800
C	-2.06428400	-0.54508700	-0.16401200
H	-2.18039600	-0.39279800	-1.24576500
C	-0.55711600	-0.55830800	0.16635000
H	-0.21718000	-1.58738400	0.31175100
H	-0.36793900	-0.03767700	1.11167900
C	-2.94760400	0.47117400	2.01416400
H	-1.96843400	0.44036200	2.49989500
H	-3.49612200	1.32697300	2.42150000
H	-3.49632300	-0.43822800	2.27214600
O	-4.13596500	0.57995600	-0.08178400
H	-4.65824000	1.27522300	0.34276600
O	0.41389600	3.76723800	-0.90592600
H	-0.24861500	3.32767600	-1.45627500
H	1.11446600	4.01222900	-1.52598700
C	3.59387300	-0.61606000	0.66634900
H	3.94174600	0.41802100	0.64246400
C	4.38685800	-1.50288700	-0.26845300
H	4.38607000	-1.11924900	-1.29086800
H	3.97341500	-2.51486000	-0.26459200
C	2.58548700	0.99906400	-1.56800700
H	2.77705100	0.33200700	-2.41716800
H	3.53620400	1.30954600	-1.13926200
H	2.03266500	1.86726100	-1.92678900
O	2.18682600	-0.60646000	0.22525100
H	1.10810900	2.62013400	0.19326600
H	0.96537100	1.69089600	1.43822800
C	3.54507500	-1.12151000	2.09039300
H	3.14837200	-2.13976000	2.12077500

H	4.55732400	-1.12666900	2.50061700
H	2.92004900	-0.47208800	2.70767100
H	5.42057400	-1.54672700	0.08257300

Product of Reaction 5C

C	1.68703200	0.36182700	-0.55740900
O	1.66125300	1.54137200	0.32273500
C	0.26224200	0.01096100	-0.96583800
H	0.31079200	-0.72694800	-1.77347300
H	-0.17685800	0.91984500	-1.39077900
C	-2.81544400	0.74383700	0.48784100
C	-2.09853500	2.04773600	0.13865800
H	-2.03974300	2.17015200	-0.94822300
H	-2.65866600	2.89392300	0.55002200
H	-1.08566500	2.07907600	0.55436500
C	-2.81087100	-1.78084500	0.21013600
H	-2.58396600	-2.09739200	1.23780500
H	-3.90388100	-1.69063300	0.16231800
C	-2.43420700	-2.92227400	-0.68323600
H	-2.91184900	-3.89099400	-0.44709200
O	-1.66381400	-2.83869300	-1.62191200
C	-2.11346400	-0.47254000	-0.15425100
H	-2.22801700	-0.33818000	-1.23868900
C	-0.60578200	-0.54023300	0.16853000
H	-0.31195500	-1.57696200	0.35467700
H	-0.40865900	-0.00704000	1.10683400
C	-2.96253600	0.61323400	2.00336900
H	-1.99232800	0.52152300	2.49950400
H	-3.45744800	1.50659600	2.39861100
H	-3.57230300	-0.25588400	2.26279600
O	-4.12138600	0.76882900	-0.10572400
H	-4.60639200	1.49851600	0.30495500
O	0.78573800	3.51983900	-0.74100600
H	0.07261200	3.27324800	-1.35562300
H	1.48802300	3.93181100	-1.27339800
C	3.53070800	-0.58348200	0.71014000

H	3.86516700	0.45302000	0.60394000
C	4.44611800	-1.51421600	-0.06541000
H	4.47671100	-1.24529100	-1.12432700
H	4.09044200	-2.54537900	0.02285700
C	2.54070100	0.77130200	-1.74375500
H	2.62547900	-0.07622000	-2.42632600
H	3.53825200	1.08584700	-1.43065600
H	2.06117500	1.60122400	-2.26961300
O	2.18870200	-0.70021500	0.18533700
H	1.19253900	2.57671100	-0.22659800
H	1.16379000	1.33164700	1.13308300
C	3.45419200	-0.93350500	2.18504400
H	3.07473500	-1.95217300	2.31144300
H	4.44542800	-0.87329600	2.64162200
H	2.78538400	-0.24357600	2.70694100
H	5.46234400	-1.46304000	0.33467500

Reactant of Reaction 5D

C	-1.74156200	0.36752500	0.23612100
O	-1.54622900	-1.98879000	0.20444600
C	-0.41756200	0.64847300	-0.38489000
H	-0.45220000	1.71554000	-0.65092400
H	-0.37473400	0.09629500	-1.32819400
C	2.79776500	-0.74421300	-0.67028600
C	1.82569400	-1.67214500	-1.39856400
H	1.40364600	-1.16922500	-2.27440000
H	2.36242100	-2.56389500	-1.73880600
H	1.00661500	-1.99925400	-0.75105400
C	3.07264400	1.51505100	0.45753900
H	3.23222300	1.19076000	1.49545500
H	4.06826100	1.53392800	-0.00386000
C	2.59716300	2.93330000	0.52066700
H	3.26348700	3.63748800	1.05169900
O	1.55660500	3.33391200	0.03235400
C	2.11777800	0.58842200	-0.29141200
H	1.88008100	1.07585100	-1.24664300

C	0.80263400	0.39029000	0.49525400
H	0.77415900	1.07409400	1.34945600
H	0.74577100	-0.62429800	0.90403000
C	3.41471000	-1.45064600	0.53608200
H	2.67997100	-1.60799500	1.33052300
H	3.80349600	-2.42900800	0.23436400
H	4.24294000	-0.86538200	0.94375600
O	3.84192500	-0.37437000	-1.58166700
H	4.33012300	-1.18082100	-1.79964000
O	0.42586000	-3.11017800	1.75391600
H	1.15428100	-3.32018200	1.15303100
H	0.78503000	-2.42381800	2.33290700
H	-0.83121000	-2.39488300	0.74141800
H	-1.30188400	-2.16656700	-0.71371100
C	-4.10575900	0.06853900	-0.23037700
C	-4.75175900	1.36947400	0.19122800
H	-4.26814900	1.79505800	1.07350600
H	-4.70572500	2.09242900	-0.62731500
C	-1.92150800	0.38840900	1.70295300
H	-2.88316300	0.00891200	2.03794000
H	-1.10856100	-0.15946600	2.17940500
H	-1.82792300	1.44308600	1.99714100
O	-2.70044100	0.34649800	-0.59934700
H	-5.80040400	1.17771500	0.43024000
H	-4.08047500	-0.66287500	0.57923700
C	-4.70969500	-0.54321900	-1.47305400
H	-5.74981800	-0.80495100	-1.26677300
H	-4.68371400	0.16880900	-2.30194100
H	-4.16956700	-1.44948400	-1.75603800

TS of Reaction 5D

C	-1.73275700	0.31849200	0.23041800
O	-1.56670400	-1.87965000	0.23944400
C	-0.40984800	0.62347600	-0.38920400
H	-0.46406000	1.68927600	-0.65480900
H	-0.35388800	0.07247200	-1.33224400

C	2.79406100	-0.76695800	-0.66958600
C	1.81259100	-1.68789700	-1.39464300
H	1.40208700	-1.18627600	-2.27661500
H	2.33855000	-2.58966100	-1.72510900
H	0.98505100	-1.99788000	-0.74892100
C	3.09161400	1.49068800	0.45564900
H	3.24564400	1.16708800	1.49464700
H	4.08833500	1.49725200	-0.00378000
C	2.63278700	2.91472200	0.51446200
H	3.30580400	3.61180000	1.04648600
O	1.59894000	3.32710500	0.02172100
C	2.12747900	0.57323800	-0.29262700
H	1.89387700	1.06239000	-1.24803200
C	0.81166100	0.38823800	0.49502900
H	0.77987200	1.09033600	1.33416700
H	0.76118300	-0.61653700	0.92712000
C	3.40717800	-1.47677900	0.53719700
H	2.67397100	-1.62638800	1.33470600
H	3.78691100	-2.45881300	0.23602600
H	4.24161300	-0.89789800	0.94138700
O	3.84035300	-0.40897800	-1.58333400
H	4.32237200	-1.21973900	-1.79893000
O	0.35851700	-3.04304800	1.76940300
H	1.08047100	-3.26581500	1.16499200
H	0.74505600	-2.39353100	2.37289000
H	-0.85207000	-2.28022900	0.78854700
H	-1.34075800	-2.10031600	-0.67513600
C	-4.09844500	0.07023500	-0.24087900
C	-4.74452200	1.37106200	0.18302300
H	-4.26071600	1.79518700	1.06581100
H	-4.69709600	2.09557900	-0.63419500
C	-1.92232700	0.39360300	1.69818200
H	-2.87382200	-0.00743600	2.03845500
H	-1.09861100	-0.10990000	2.20319500
H	-1.87148900	1.46153200	1.95009100
O	-2.69668900	0.34409600	-0.61000500

H	-5.79373900	1.18137500	0.42148800
H	-4.07822900	-0.66204500	0.56836800
C	-4.70903900	-0.53861500	-1.48255800
H	-5.75058900	-0.79483800	-1.27639200
H	-4.67969000	0.17326200	-2.31162600
H	-4.17398300	-1.44747100	-1.76718800

Product of Reaction 5D

C	-1.64166800	0.08880500	0.23328900
O	-1.63524100	-1.37463900	0.21475900
C	-0.33824600	0.61108400	-0.35862000
H	-0.46350600	1.68286900	-0.54201700
H	-0.22009500	0.13316000	-1.33780800
C	2.79160400	-0.92925900	-0.62106800
C	1.75544400	-1.79785600	-1.33376700
H	1.38634300	-1.29346500	-2.23183300
H	2.21950100	-2.74296800	-1.63473800
H	0.90518900	-2.03148600	-0.68478900
C	3.24346800	1.33382800	0.43995700
H	3.37590700	1.03388700	1.48899600
H	4.23862700	1.25911200	-0.01720600
C	2.88234700	2.78717800	0.45322100
H	3.59772200	3.45230300	0.97106400
O	1.88289500	3.25446600	-0.06094500
C	2.21741300	0.46283100	-0.28059000
H	2.01166400	0.94090600	-1.24797100
C	0.89696800	0.38345800	0.51481900
H	0.90242200	1.13066100	1.31502700
H	0.82869200	-0.58796000	1.01462100
C	3.34020700	-1.65996800	0.60452400
H	2.57193300	-1.80441000	1.37037500
H	3.70718900	-2.64779300	0.30669500
H	4.17254500	-1.10757600	1.04802700
O	3.87031200	-0.66870500	-1.52989100
H	4.27787200	-1.52029300	-1.74184500
O	-0.44762200	-2.68175400	1.85662100

H	0.47800100	-2.82175300	1.58900400
H	-0.43030300	-2.21495900	2.71044000
H	-0.98042100	-2.04108700	1.08649300
H	-1.43087400	-1.68292600	-0.68554100
C	-4.00519800	0.11507600	-0.34357800
C	-4.77449900	1.33516900	0.13309100
H	-4.33476400	1.74881500	1.04418000
H	-4.76224500	2.10834900	-0.64137200
C	-1.86653100	0.48355400	1.68066100
H	-2.82270900	0.10903700	2.05101100
H	-1.07100500	0.07793900	2.31031200
H	-1.85291500	1.57324000	1.75515800
O	-2.64688500	0.50334400	-0.64215300
H	-5.81477300	1.06957900	0.34003700
H	-3.99410600	-0.65451300	0.43464600
C	-4.58394500	-0.47529900	-1.61693900
H	-5.62280200	-0.77593400	-1.45846400
H	-4.55615600	0.26556700	-2.42194600
H	-4.00880800	-1.35160200	-1.92769700
